# Thermal Conversion of Coal Bottom Ash and Its Recovery Potential for High-Value Products Generation: Kinetic and Thermodynamic Analysis with Adiabatic *T_D24_* Predictions

**DOI:** 10.3390/ma17235759

**Published:** 2024-11-25

**Authors:** Bojan Janković, Marija Janković, Ana Mraković, Jelena Krneta Nikolić, Milica Rajačić, Ivana Vukanac, Nataša Sarap, Nebojša Manić

**Affiliations:** 1 “Vinča” Institute of Nuclear Sciences—National Institute of the Republic of Serbia, University of Belgrade, Mike Petrovića Alasa 12-14, P.O. Box 522, 11001 Belgrade, Serbia; bojan.jankovic@vinca.rs (B.J.); amrakovic@vinca.rs (A.M.); jnikolic@vinca.rs (J.K.N.); milica100@vinca.rs (M.R.); vukanac@vinca.rs (I.V.); natasas@vinca.rs (N.S.); 2 Faculty of Mechanical Engineering, Fuel and Combustion Laboratory, University of Belgrade, Kraljice Marije 16, P.O. Box 35, 11120 Belgrade, Serbia; nmanic@mas.bg.ac.rs

**Keywords:** bottom ash conversion, valuable materials, thermodynamics, reaction mechanism, autocatalysis, adiabatic conditions

## Abstract

Thermal decomposition (pyrolysis) of coal bottom ash (collected after lignite combustion in coal-fired power plant TEKO-B, Republic of Serbia) was investigated, using the simultaneous TG-DTG techniques in an inert atmosphere, at various heating rates. By using the XRD technique, it was found that the sample (CBA-TB) contains a large amount of anorthite, muscovite, and silica, as well as periclase and hematite, but in a smaller amount. Using a model-free kinetic approach, the complex nature of the process was successfully resolved. Thermodynamic analysis showed that the sample is characterized by dissociation reactions, which are endothermic with positive activation entropy changes, where spontaneity is achieved at high reaction temperatures. The model-based method showed the existence of a complex reaction scheme that includes two consecutive reaction steps and one single-step reaction, described by a variety of reaction models as nucleation/growth phase boundary-controlled, the second/*n*-th order chemical, and autocatalytic mechanisms. It was established that an anorthite *I*1 phase breakdown reaction into the incongruent melting product (CaO·Al_2_O_3_·2SiO_2_) represents the rate-controlling step. Autocatalytic behavior is reflected through chromium-incorporated SiO_2_ catalyst reaction, which leads to the formation of chromium(II) oxo-species. These catalytic centers are important in ethylene polymerization for converting light olefin gases into hydrocarbons. Adiabatic *T_D24_* prediction simulations of the process were also carried out. Based on safety analysis through validated kinetic parameters, it was concluded that the tested sample exhibits high thermal stability. Applied thermal treatment was successful in promoting positive changes in the physicochemical characteristics of starting material, enabling beneficial end-use of final products and reduction of potential environmental risks.

## 1. Introduction

Coal is the energy source with the highest percentage of representation among estimated fossil fuels (over 65%), with more evenly distributed deposits in the world when compared to oil and gas deposits. The biggest coal reserves are located in Russia, USA, China, Australia, and South Africa, and the biggest coal reserves in Europe are in Germany, Poland, Czech Republic, and Great Britain. Coal is a widely distributed fossil fuel in the world. About 23% of the need for primary energy and 39% for electricity is obtained on the basis of coal. Due to stock reduction in oil and gas, a significant increase in the use of coal is expected in the future [1]. Today, about 40% of the total energy in the world is produced from coal, and it is expected, considering the energy projections, that this situation will remain in the future. Regulations regarding environmental protection require a reduction in CO_2_ emissions *per* megawatt produced [2]. Lignite is a mineral raw material that is most rationally used as energy fuel for energy production [3]. Due to its application and quantity, it represents an exceptional potential in energy meaning. It is brown–black in color and has a high moisture content, which sometimes reaches 66%, and it also has a high ash content compared to bituminous coal. Due to its low energy density, lignite is not cost-effective to transport and is not traded on world markets, as is the case with other types of coal that have a higher carbon content. It is often burned in thermal power plants that are built near lignite mines [4].

Bottom ash from thermal power plants is an industrial by-product (waste material) that is produced during the burning of coal. Since coal represents the mineral raw material, it can be divided into combustible and non-combustible parts. The combustible part consists of a solid organic substance and volatiles, while the non-combustible part consists of moisture and ash. In addition to the fly ash, which is represented by about 85–95% of the total amount of ash from thermal power plants, the bottom ash is represented by about 10% [5]. The annual production of fly ash in Serbia is about 6 million tons and bottom ash is 500–600 thousand tons. From the point of view of chemistry, ash is defined as the solid, non-combustible residue that remains after coal has been ignited. In industrial practice, the term ash means a solid, non-combustible residue that is separated from the combustion of coal in boilers. Several types of ash can be distinguished: (a) bottom ash—the largest non-combustible combustion residue that is separated at the bottom of the boiler; (b) boiler ash—larger classes that are separated from the boiler together with flue gases but on the way to the electric filter, they settle by gravity and are separated under the flue gas channel, and under the air heater; (c) electro-filter or fly ash—the smallest classes that come out of the boiler separated with flue gases, and separation from the stream of flue gases are carried out by electrostatic separation in electro-filters or in bag filters. One of the problems that accompany the production of electricity from coal is the problem of the so-called “energy waste”. The term energy waste means ash and bottom ash (also ‘slag’), which are formed as incombustible and unburnt residues during coal combustion and waste (most often gypsum) obtained by desulfurization of flue gases [6]. Quantities of energy waste in the world are allocated annually in excess of one billion tons. At the same time, ash and slag are characterized as inert, hazardous, and non-hazardous waste, while gypsum is classified as non-hazardous waste. So far, only a small part of fly ash (up to 10%) and a very small part is in Serbia, where the amount of bottom ash found its application, while the rest is disposed of in nearby landfills and thermal power plants, which cover significant areas of arable land, presenting a very large technical, economic, and environmental problem. The bottom ash has been used to a lesser extent in the brick industry, in the reconstruction and construction of low-level roads, as well as in the production of “cinder blocks”. However, in the European Union (EU) member states, the bottom ash is intensively and increasingly used for landfill encapsulation.

Coal and the coal combustion ash represent alternative and unconventional resources for critical materials because many coals already contain a significant amount of concentrated valuable metals hosted in coal partings, and coal minerals as fine-grained mineral relics, the neo-formed minerals, and aluminosilicate glasses in the coal combustion ash [7]. Metal concentrations in combustion coal products (CCP) are available in high amounts due to their original concentration in coal-forming basins and to the large volumes of coal burnt to satisfy the electricity demands, generating huge amounts of organic matter-free mineral phases [8]. The quantity of coal bottom ash produced in the thermal power plant depends on the amount of coal burned, combustion conditions, and the amount of mineral and other metal compounds in the coal. The significant quantities of minerals and metals present in residues offer various opportunities for re-utilizing. The utilization of CCP is reflected in cement-based materials, construction applications, mining applications, geotechnical and agricultural purposes, waste stabilization, and many others [9,10,11]. Using CCP conserves national resources then, saves energy, reduces the associated carbon emissions, as well as contributes to an efficient circular economy [12]. In other words, we can express the CCP as shifting from waste disposal to resource recovery.

Based on the physical properties that were studied previously [13,14], the coal bottom ash has been identified to have a specific gravity that ranges between 1.39 and 2.47, which categorizes it as less dense than the typical value of natural sand (~2.65); however, the density of coal bottom ash still depends on how the material was processed. In addition, coal bottom ash samples that undergo metal recovery (ferrous and non-ferrous metal exclusion) and pre-treatment (washing) showed that their specific gravity was 2.20 due to the elimination of metal elements, such as aluminum (Al), copper (Cu), iron (Fe), and lead (Pb). Different specific gravity values reported for coal bottom ash could be influenced by additional mechanical treatment, especially grinding some particles into a smaller size [15]. The main oxides present in coal bottom ash are as follows: SiO_2_ (45.30–59.82%) and Al_2_O_3_ (17.65–27.76%), while Fe_2_O_3_ (3.77–19.84%), K_2_O (1.29–3.48%), CaO (0.75–8.70%), and MgO (0.40–3.13%) were also identified but in the smaller quantities. The coal bottom ash owns a range of loss of ignition (LOI) between 0.89–8.10. The high LOI value, as influenced by its organic compounds and other present impurities, such as unburned carbon, can compromise the structural integrity and mechanical performance of the material [16].

Recovery of metals from such waste and its utilization is important, not only for saving the metal resources but also for protecting the environment. Recovery can be implemented through the chemical route by extraction and leaching available procedures and by subsequent thermal treatment (via pyrolytic processing) with raw material (which suffered burnout) using thermal analysis techniques [17].

The main goal of this work is the assessment of the recovery potential of coal bottom ash obtained in the coal-fired power plant “Kostolac B” (TEKO-B) in the Republic of Serbia, as the residue (waste) product of lignite combustion (TEKO-B landfill for disposal of ashes occupies a large area, about 600 ha). On the one hand, this assessment was carried out on the basis of chemical and structural analysis of the raw material, where an appropriate conclusion was drawn. The other side covers the assessment of recovery potential through kinetic and thermodynamic analyses of the thermal decomposition process of coal bottom ash in an inert atmosphere, which allows elucidation of reaction mechanisms and energy pathways leading to the identification of valuable end-products. Additionally, two kinetic approaches were used: the model-free (isoconversional) and model-based (model-fitting) calculation approaches. Broadly speaking, methods for processing non-isothermal thermogravimetry (TG) data can be categorized into model-fitting and model-free methods. Namely, the first type requires the assumption of reaction models or mechanisms and then manipulating the experimental data based on the selected models. The model-free methods, comparatively, require no assumption of reaction models during the data operation.

In order to make a thermal risk assessment in analyzed chemical processes, the adiabatic prediction, called *T_D24_* analysis, was implemented. In this paper, the actual approach was applied for the first time to examine the process of coal bottom ash conversion in terms of safety for industrial purposes, considering the least possible impact on the environment and reducing pollution. Lack of knowledge about the process may lead to incorrect process conditions and thus to thermal runaway in equipment or reactors. For the latter, the specific temperature called the maximum temperature of synthesis reaction (MTSR) is essential for assessing the thermal runaway risk and designing safe operating conditions. The applied prediction that was made in this study is useful if the process takes place at an industrial scale stratum, inside the large reactors, whereby reactants undergo conditions close to the adiabatic, and where evolving heat energy leads to self-heating of reactants. The initial temperature for an adiabatic process with time to the maximum rate (TMR) is equal to 24 h, and therefore, it was called *T_D24_*. This corresponds to the temperature at which the time to maximum rate of runaway reaction is 24 h. Thus, this temperature characterizes the process, and it is commonly used for thermal risk assessment. This work provides new insights into the sustainable application of coal bottom ash while having as its ultimate goal a high-value application, which includes energy catalysis, valuable metals recovery, and material synthesis.

## 2. Materials and Methods

### 2.1. Material

Coal bottom ash (CBA) was collected in the coal-fired power plant “Kostolac B” (TEKO-B) in the Republic of Serbia, and it was obtained from the combustion process of lignite coal. To withdraw moisture and facilitate its milling, the bottom ash went through oven drying at *T* = 105 °C for 24 h. After the drying stage, the bottom ash was milled in a ball mill, model CS-501 K, for 24 h under a rotation speed of 65 rpm and with alumina balls of 10 mm. The prepared powder sample was designated as the CBA-TB sample, and this label will be used further in the text.

### 2.2. Chemical and Structural Characterization

Chemical composition (identification of present oxides) analysis and heavy metals content of the CBA-TB sample were determined using the wavelength-dispersive equipped X-ray fluorescence (WD-XRF) spectroscopy (Oxford WD/ED-2000 XRF, Oxford Instruments, Abingdon, Great Britain) and inductively coupled plasma mass spectrometry (ICP-MS) (Thermo Scientific iCAP 6000 ICP spectrometer, Thermo Fisher Scientific, PPD, Inc., Waltham, Massachusetts, United States of America). To identify the presence of crystalline phases, the X-ray diffraction (XRD) technique was performed. The X-ray powder diffraction (XRPD) analysis of the CBA-TB sample was done on the Rigaku SmartLab (Rigaku Corporation, Tokyo, Japan) X-ray diffractometer to identify the bottom ash mineralogy content. The diffraction pattern of CBA-TB was recorded for the phase analysis in the 10–80° 2θ range, with the step size and exposition of 0.01° and 2° *per* minute, respectively. The 2θ correction was 0.05°. The XRD pattern was collected by measuring the scintillation response to Cu Ka radiation at a wavelength of λ = 1.541874 Å against 2θ value for the considered 2θ—range. The X-ray powder diffraction and semi-quantitative analysis of the CBA-TB sample were performed using a program called “Match!” (CRYSTAL IMPACT, Bonn, Germany), which was provided by the Institute of Technical Science of SASA (Serbian Academy of Sciences and Arts). For the prepared bottom ash sample, any treatment prior to analysis measurement was not required. Namely, the CBA-TB sample was spun during the data collection in order to obtain the best peak profile and minimize a preferred orientation effect.

### 2.3. The Radiochemical Characterization

For the gross alpha/gross beta activity and for gamma spectrometric measurement, sample preparation includes drying at the temperature of 105 °C. For the gross alpha and gross beta activity measurement, about 130 mg was weighed in an aluminum planchet and fixated with alcohol [18]. For gamma spectrometric measurement, the sample was placed into the Marinelli beakers and sealed with bee wax. For the purpose of achieving the radioactive equilibrium, the sample was left in the laboratory for 30 days prior to the measurement [19].

The low-level proportional counter Thermo-Eberline FHT 770T was used for the gross alpha/gross beta activity measurement. Counting efficiencies were determined using the certified radioactive calibration standards ^241^Am and ^90^Sr (9031-OL-334/11 and 9031-OL-335/11, respectively, Czech Metrology Institute). The counting efficiency was 26% for alpha and 35% for beta radiation, respectively. The measurement time was 14,400 s.

For gamma spectrometry measurement, the High-Purity Germanium (HPGe) detector (AMETEK ORTEC, Nuclear Instrumentation, AMETEK, Inc., Berwyn, Pennsylvania, United States of America) with a relative efficiency of 18% was used. The calibration of the detector was performed using the certified radioactive standard (1035 SE-40845-17, Czech Metrology Institute, Inspectorate for Ionizing Radiation) spiked with ^241^Am, ^109^Cd, ^139^Ce, ^57^Co, ^60^Co, ^137^Cs, ^113^Sn, ^85^Sr, ^88^Y, ^51^Cr, ^210^Pb, and ^113^Sn with total activity of 80.6 kBq. The measurement time was 60,000 s. In addition, the measurement results were expressed as Bq/kg with the confidence level of 95% (k = 2).

### 2.4. Thermal Decomposition Analysis Using the Simultaneous Thermogravimetry (TG) and Derivative Thermogravimetry (DTG) Measurements

Thermal decomposition measurements were performed in non-isothermal conditions, using NETZSCH STA 449 Jupiter F5 simultaneous thermal analysis device (Die Netzsch Gruppe, Group - NETZSCH Holding, NETZSCH-Vertriebs-Gesellschaft mbH, Selb, Germany), in an argon (Ar) atmosphere (carrier gas purity was Ar Class 5.0). Thermal analysis experiments were implemented at the heating rates (*β*) of *β* = 10.3, 20.9, and 32.1 K/min, with a mass of a powdered sample of approximately Δ*m_sample_* ≈ 8.0 ± 0.1 mg, using the gas flow rate of *φ* = 40 mL/min, and within the experimental temperature range of Δ*T* = 40–800 °C. The linear programmed heating was employed for every considered thermo-analytical (TA) measurement. Simultaneously with a recording of the thermogravimetry curve for each heating rate applied, the corresponding derivative thermogravimetry (DTG) curve was displayed through the NETZSCH Proteus^®^ software (Windows® 7, 32-/64-bit, Professional, Enterprise and Ultimate; Windows® 8.1 Pro and Enterprise; Windows® 10 Pro and Enterprise, version 6.1.0, 2024). The duplicate non-isothermal runs were performed under the same conditions, and the data were found to overlap with each other (including the control measurement for each heating rate used, with approximately the same mass of the sample), indicating satisfactory reproducibility.

## 3. Model-Free and Model-Based Kinetic Analysis with the Process Simulations

The isoconversional (model-free) methods developed by Friedman (FR), Kissinger-Akahira-Sunose (KAS), Ozawa-Flynn-Wall (OFW), and Vyazovkin (VY) were applied to calculate the activation energy (*E_a_*) for the CBA-TB thermal decomposition process under non-isothermal conditions. Values of the logarithm of pre-exponential factors (log*A*) were obtained by these four methods from the application of the kinetic compensation effect (KCE). Since there was no significant difference between the values of kinetic parameters estimated from indicated methods, while Vyazovkin’s and Friedman’s methods present generic mathematical advantages in that context, the values determined by these methods were used (one of these methods was taken, which gives a better statistical comparison of the results in terms of fitting qualities, with those obtained by the experimental procedure) to find the best fit, among the reaction models described in the Supplementary Materials (Section S.II.). Also, the theoretical backgrounds of model-free methods used in this work are presented in the Supplementary Materials (Section S.I.). Based on the received model-free (isoconversional) data from Friedman’s (FR) method, the numerical optimization (using the non-linear least square (NLLS) optimization) of the investigated process was applied, and this procedure is explained in the Supplementary Materials (Section S.I.) [20,21,22,23,24,25,26,27,28,29,30,31]. In addition to these methods, the model-based approach was applied to the considered thermal decomposition process. More details about this kinetic approach were given in the Supplementary Materials (Section S.II.) [32]. All kinetic calculations were carried out using the NETZSCH Kinetics Neo software (Product version 2.7.3.15, Build date 6 November 2024) [33]. On the other hand, for a theoretical description of the applied adiabatic prediction simulations, the reader is referred to Supplementary Materials (Section S.III.) [34,35,36,37,38,39,40,41,42].

## 4. Results and Discussion

### 4.1. Chemical Characteristics of CBA-TB

Chemical characteristics of the bottom ash are mainly determined depending on the type and quality of the coal source, not by the pulverized fineness and operating conditions of the coal-fired power plant. Chemical composition and heavy metals (HMs) were analyzed using WD-XRF and ICP-MS techniques. Table 1 lists the percentage contribution (the content in percentages [%]) of basic components in the CBA-TB sample, determined by the WD-XRF spectroscopy, as well as the content of heavy metals (HMs), determined by the ICP-MS technique. Table 1 compares the mineralogical composition and LOI of the CBA-TB sample with those reported in the literature [13,43,44,45].

It can be seen from Table 1 that the tested sample primarily consisting silica (SiO_2_), alumina (Al_2_O_3_), and ferric oxide (Fe_2_O_3_), which account for more than 75% of the total components. However, calcium oxide (CaO), magnesia (MgO), and sulfur trioxide (SO_3_) are also present in a higher proportion, and they are typical for the coal bottom ash produced from lignite combustion [46]. This mineral composition is very similar to the fly ash [47,48,49]. The bottom ash and fly ash, which were characterized by a relatively high content of CaO, can have the latent hydraulic property, which may slightly enhance the cohesion between particles. The higher content of CaO and Fe_2_O_3_ in the CBA-TB sample (Table 1) suggests an increased intensity of the bottom ash (increased content of CaO would result in a lower melting temperature of considered bottom ash). So, the increased content of CaO may reduce the degree of structural complexity of the reacted system.

Referring to Table 1, it can be noted that CBA-TB can be classified as ASTM C 618 by the Class F ash, with the percentage combination of SiO_2_, Al_2_O_3,_ and Fe_2_O_3_, ranging between 70% and 93%, which exceeds the value of 70% (for our sample, this amounts 79.23%) [50,51,52]. Loss on ignition (LOI) (in Table 1, it is referred to as the loss to fire) for the bottom ash ranged from 1.80 to 3.99, and this can be attributed to the amount of carbon to measure the quantity of carbon dioxide (CO_2_) [53]. Considering the mentioned facts, the coal bottom ash with its properties represents the alternative for the cement additions, as the partial alternative of Portland cement (PC) in concrete [54,55].

With respect to the utilization of the bottom ash, the amount of heavy metals (HMs) must be considered because bottom ash is often considered not harmful. The reactivity and potential of heavy metal released by the reaction of bottom ash with CO_2_ and water will further reduce as time passes through the aging and weathering process, which results in more stable complex compounds in the bottom ash [56]. From the results presented in Table 1, among the heavy metals (HMs), Cr, Ni, Cu, Ba, and As are the most abundant, where the CBA-TB represents a chromium-enriched bottom ash, whereby Cr is the environmentally sensitive element. In addition, the fate and distribution of these elements primarily depend on the mode of the occurrence of these elements in the coal and mineral phase transformation at different temperatures of the combustion process [57]. Because the CBA-TB sample contains a very high concentration of chromium, the CBA-TB must be taken with caution, so some effort should be made regarding the treatment of such material to prevent the possible leakage of Cr into surrounding waters and soils. According to the International Agency for the Research on Cancer, chromium and nickel (compounds as group 1) have carcinogenic effects on humans [58]. An increased concentration of As should be emphasized (Table 1) since the high-temperature combustion process may lead to the oxidation of As(III) or As(0) into As(V) [59]; the presence of calcium (Ca) can play an important role in an interacting with arsenic vapor in the slow solid/gas surface reaction mechanism, specifically in the post zone. Considering these analyses, the CBA-TB would require the implementation of the chemical extraction procedure for the mentioned metals to significantly reduce potential environmental risks. One of the immobilization procedures for these toxic elements would be the thermal deconstruction of the coal bottom ash into compounds that would reduce or cancel their toxic action and, at the same time, recycle it toward needed (critical) materials. Therefore, the coal bottom ash would have great potential to produce value-added materials.

### 4.2. Results Related to the Radiological Characterization of CBA-TB

In the CBA-TB sample, the naturally occurring radionuclides ^226^Ra, ^232^Th, ^40^K, ^235^U, and ^238^U were detected (obtained activity concentrations are given in Table 2). The activity concentration of artificial radionuclide ^137^Cs was below the minimum detectable concentration. The activity ratio of ^235^U/^238^U corresponds to natural uranium in the sample. Values obtained for gross alpha activity and gross beta activity were below the minimum detectable concentration (Table 2). Table 2 also contains a comparison of some radionuclides present in the coal bottom ash, which have been reported by other researchers [60,61].

The gross alpha activity in samples originates from the decay chains of ^238^U and ^232^Th, while the gross beta activity originates from long-lived radioisotopes, such as ^40^K, ^210^Pb, and ^228^Ra. Considering that the bottom ash is produced as the by-product during the coal combustion process, natural radionuclides are concentrated in the bottom ash. The obtained values of the natural radionuclides ^226^Ra, ^232^Th, and ^40^K in the bottom ash are 2.4 times and 1.4 times higher compared to the lignite coal, while the uranium values are at the same level.

In order to assess whether the tested bottom ash can be safely used, for example, in the building construction material, the radium equivalent activity and the external hazard index can be determined based on the obtained activity concentrations of naturally occurred radionuclides ^226^Ra, ^232^Th, and ^40^K, in the CBA-TB sample [62]. The calculated value was 71 Bq/kg for radium equivalent activity, and this result is lower than the safety limit of 370 Bg/kg. The calculated value for the external hazard index was 0.2 Bq/kg. The hazard index should not exceed 1, and in that case, the radiation risk from the gamma radiation is negligible. The obtained results were compared with the results for coal bottom ash from the other coal-fired power plants [60,61]. The values of ^226^Ra, ^232^Th, and ^40^K obtained in this paper are lower than the results given in the above-mentioned references [60,61]. The disagreement in activity concentration values between coal ashes is due to the great variability of the activity concentration of coals around the world. These results are in good agreement with the requirements needed to consider potential health risks [63]. Consequently, the tested sample is classified as those products that do not have any restrictions for further use. Thus, in the observed case, the CBA-TB does not represent a danger to the public health in any case and therefore, can be used in the construction as building material.

### 4.3. XRD Analysis of CBA-TB

The XRD pattern together with the complete “Match!” report related to the main crystalline phases detected in CBA-TB are presented in Figure S1 (Supplementary Materials). It can be seen from Figure S1, that the CBA-TB sample contains approximately 50% of feldspar-group mineral, anorthite (CaAl_2_Si_2_O_8_), and less than this are aluminosillicates of potassium (approximately 30%) and muscovite. In addition, the rest of the structural phases present in the CBA-TB sample represent the quartz (SiO_2_) (about 20%) (the ratio of SiO_2_ in a bottom ash is usually a higher from the one present in fly ash, most likely because of the presence of the glass in the waste raw material), hematite (Fe_2_O_3_) (about 3%), and the periclase (MgO) (~3.5%) (Figure S1). The presence of anorthite is characteristic of only the coal bottom ash, but the presence of both, hematite (Fe_2_O_3_) and quartz (SiO_2_) are characteristic of bottom ash and fly ash [64]. Also, the amorphous phase can be detected in the CBA-TB sample (Figure S1), along with high intensity crystalline peaks of the quartz and K-aluminosillicate. The amorphous region arises from unburned residual carbons in the tested sample (namely, it can be seen a wider diffraction peak in the vicinity of 24° (2θ) (Figure S1), which originates from the amorphous carbon that was present in the sample). Based on the obtained XRD data, the considered CBA-TB sample can be characterized as the bottom ash with high amounts of silica, alumina, and iron contents, with particle fineness and some degree of amorphousness. However, for a more detailed analysis regarding this, it should be considered that the total amount of the three oxides related to silica oxide, aluminium oxide, and iron oxide must be greater than 70% [65], and this condition is met for our investigated sample (CBA-TB), considering the results presented in Table 1.

### 4.4. TG-DTG Analysis of CBA-TB Thermal Conversion Process

**Figure 1:**
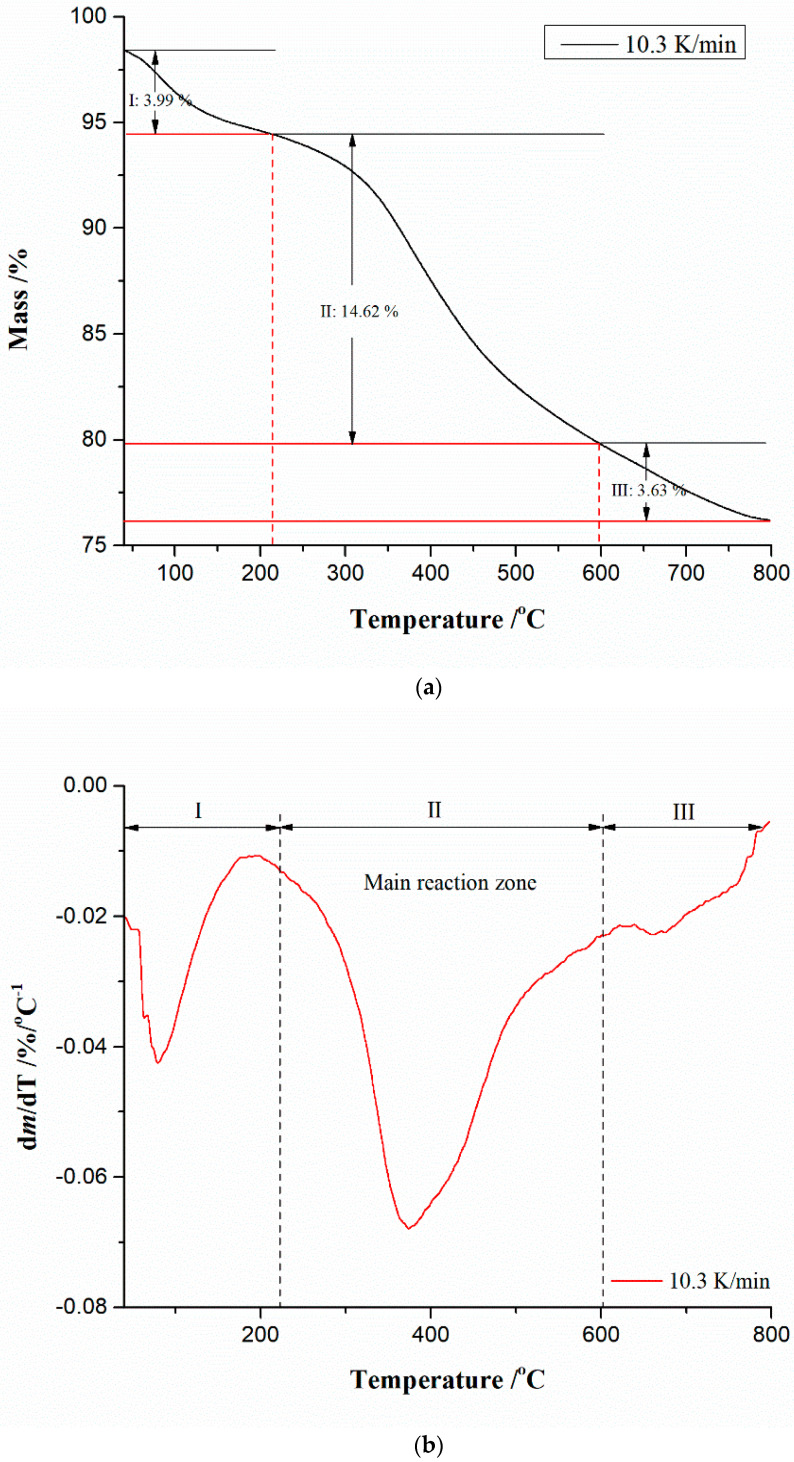
TG (**a**) and DTG (**b**) curves at the heating rate of *β* = 10.3 K/min for thermal decomposition of CBA-TB.

Diagrams of thermogravimetry (TG) and derivative thermogravimetry (DTG) analyses in the thermal conversion process of the CBA-TB sample in Ar atmosphere at *β* = 10.3 K/min are shown in Figure 1a,b.

Thermal decomposition reactions are usually endothermic because heat is needed to break the chemical bonds in the considered compound. The reduced mass can also provide information about the thermal stability of the material and kinetic parameters of chemical reactions that occur within the sample. From both thermal analysis features (Figure 1a,b), three zones of the decomposition process can be observed. The first one (“I”), is characterized by a mass loss of ~3.99% (Figure 1a), which can be attributed to the moisture elimination from the sample because this class of ash is very sensitive regarding moisture where it exhibits great affinity for absorbing the moisture, through the small framework pores [66]. This is followed by a stage (the second one, designated by “II”) with a greater mass loss of the sample (~14.62%) and represents the main processing (reaction) zone (Figure 1a,b), so it is obvious that some compounds present in the CBA-TB undergo thermal decomposition. Beyond 600 °C, it can be observed the existence of the DTG peak on the side of the high temperatures (Figure 1b), where a certain decomposition reaction (“III”) of the compound occurs, which is characterized by the mass loss of the sample of ~3.63% (Figure 1a). The two zones, “II and III”, represent two main reaction areas during the CBA-TB thermal decomposition process. The residual mass of the sample, after the thermal decomposition, amounted to Δ*m_res_*~76.20%. Based on the obtained thermal analysis patterns, it is obvious that operating temperatures have a significant effect on the content and thermal properties of CBA-TB, which can be attributed to the chemical diversity of minerals contained in the investigated sample.

It should be emphasized that the thermal analysis of CBA-TB provides important information on its reactivity and “hardening”, which is necessary for the effective utilization of this type of coal bottom ash. Namely, the quartz and alumina components of the coal are transformed in their crystalline states at high temperatures, but if there is a crystalline phase such as Fe_2_O_3_, only 20% to 30% of the coal bottom ash is in a crystalline state, and the rest is usually present in the glassy state. Namely, the oxides of Al and Fe with a higher boiling point may be present in the bottom ash, in opposition to the oxides of K and Na, which are primarily present in the fly ash having lower boiling points. Except for the quartz, which does not undergo chemical deformation at high temperatures and instead mostly retains its original shape, most of the minerals in the coal are generally broken down during the combustion.

**Figure 2:**
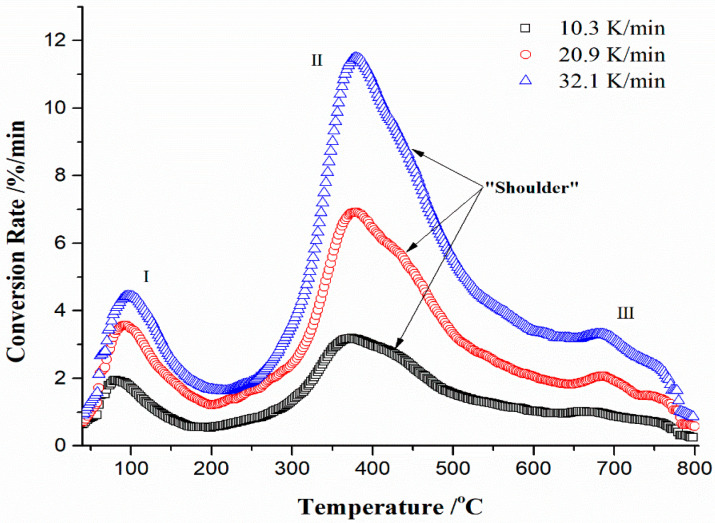
The conversion rate (absolute) (%/min) curves vs. temperature at various heating rates for thermal decomposition of CBA-TB (the specific decomposition zones are also designated at the same graph, as “I, II and III”; the corresponding “shoulder” reaction peak inside the main decomposition region (“II”) is clearly marked).

Thermo-analytical (TA) traces shown in Figure 1a,b generally reflect the thermal stability of a given sample, identifying that there is some decomposition of the mineral components in the ash, but it cannot be stated with the precision, in which temperature interval a certain component is decomposed, without additional kinetic analysis of the process. Figure 2 shows the absolute conversion rate (expressed in %/min) curves at the different heating rates against the temperature, for the thermal decomposition of the CBA-TB sample.

The absolute conversion rate curves at various heating rates in a given experimental temperature interval are trustworthy and reflect the derivative-TG runs, confirming the existence of reaction zones in the entire decomposition, which were indicated above. However, within the main zone decomposition for Δ*T* ≈ 225–550 °C, there is an existence of a “shoulder” reaction peak, indicating a more complex kinetics event, which takes place in this zone. Therefore, the shapes of the conversion rate curves clearly show that the process of CBA-TB thermal decomposition does not contain simple reactions that would be explained by modest kinetic models. It is possible that the kinetics of the process becomes more complicated, whereby the effect of reactions overlapping in the given temperature regions can come to the fore.

Table 3 lists the values of maximum peak temperatures (*T_p_*) attached to the maximum reaction rates (*R_max_*) in regions I, II, and III, respectively, at different heating rates (*β* = 10.3 K/min, 20.9 K/min and 32.1 K/min).

Considering the influence of heating rate, both *R_max_* and *T_p_* for all regions increase with an increase in heating rate, where the smallest increment between *T_p_* values regarding *β*’s is noticed for region II. By the lateral behavior of characteristic parameters shown in Table 3, it can be noticed that the highest heating rate (32.1 K/min) pushes the reaction within the observed region towards higher temperatures (*T_p_*-value), while independent of the heating rate, the highest conversion rate of the entire process is achieved in the region II. Consequently, from these observations, it can be concluded that actual conversion represents a strongly temperature-dependent process, which can directly affect the magnitude range of kinetic parameters, such as activation energy and the pre-exponential factor.

**Figure 3:**
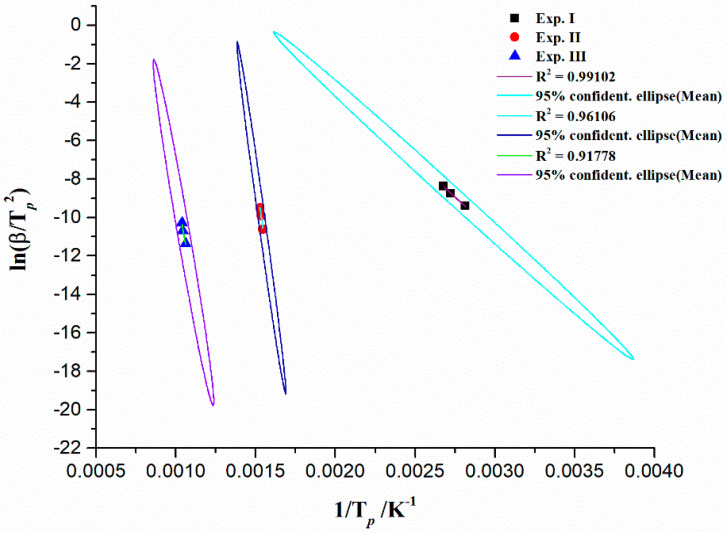
ASTM E2890 plots were obtained for considered reaction regions (I, II, and III) in thermal decomposition of CBA-TB sample (Adj. R-Square values (R^2^) and the 95% confidential (mean) ellipses are provided).

Based on data provided by TG measurements at different heating rates, we applied theoretical methods such as ASTM E2890-12 [67] based on first-order kinetics that allow the determination of activation energy (*E_a_*) and pre-exponential factor (*A*) using *T_p_* values for all identified decomposition regions (Table 3). The plot of ln(*β*/*T_p_*^2^) vs. 1/*T_p_* gives the straight line, where from the slope and the intercept, the appropriate values of *E_a_* and *A* can be calculated. Since decomposition reactions are usually multi-step and not first-order reactions, this approach provides only the general values of kinetic parameters in a single point of thermo-analytical (TA) signal. Corresponding linear plots attached to decomposition regions I, II, and II are shown in Figure 3. For all identified decomposition reaction regions, the confidence regions were constructed, which are characterized by ellipses for the least squares estimates of kinetic parameters in the case of the linear regression procedure within the standard test kinetics method.

Table 4 shows the values of kinetic parameters (the activation energy (*E_a_*) and pre-exponential factor (*A*)) for separated reaction regions (Figure 3), in an entire decomposition process of CBA-TB, obtained by the standard method.

It can be seen from Table 4 that both kinetic parameters (*E_a_* and *A*) exhibit strong variation with respect to different decomposition regions, reflecting complex intrinsic kinetics (not as simple consideration), going from one reaction zone to another. From these results, the corresponding fixed conversion level at *T_p_* should be independent on the variation of *T_p_* with a variable heating rate extent for a simple first-order reaction, which was assumed here. However, in this case, it is the opposite, indicating that the given process cannot be explained by the simple kinetics, such as zero- or first-order reactions. This observation can be identified from the estimated magnitudes of the pre-exponential factor (*A*). Namely, the pre-exponential factor is the pivotal parameter which reflects the surface structure of investigated sample or the complexity of reactions during thermal conversion. When the magnitude of *A* is less than 10^9^ s^−1^, the reaction represents the surface reaction, while the magnitude of *A* is greater than or equal to 10^9^ s^−1^, it means an emergence of complex reactions [68]. It can be seen from Table 4 that the magnitude of pre-exponential factor varies in the range between ×10^7^ s^−1^ (region I) and ×10^37^ s^−1^ (region II) (including also the region III with magnitude of *A* of × 10^19^ s^−1^) so these results clearly indicate that thermal decomposition of CBA-TB involves multiple parallel reactions, which are very complicated, and they cannot be described by the simpler kinetics. Namely, high values of both *E_a_* and *A*, especially within region II (Table 4), can indicate on the existence of synergistic interaction of chemical components found in the CBA-TB sample, which may lead to an increase in the activation energy and pre-exponential factor.

In this study, the thermodynamic parameters of activation, such as changes in the activation enthalpy (ΔH*) and activation entropy (ΔS*), as well as the change in Gibbs free energy of activation (ΔG*), were calculated on the basis of conventional equations from the theory of transition state (the thermodynamic functions of activated complexes) [69]. These equations are well-established, and they can be found elsewhere [69,70]. The mentioned thermodynamic expressions are adopted for the peak reaction temperature (*T_p_*—Table 3), and this temperature, theoretically, represents the temperature at which the chemical reaction should proceed spontaneously from the point of view of equilibrium thermodynamics (namely, peak temperatures are kinetically controlled and coincide with the maximum decomposition reaction rates (Figure 2 and Table 3)). Considering ΔH*, the difference in energy between the feed material and the activated complex is determined by ΔH*. In addition, the ΔH* value determines whether a chemical reaction is endothermic or exothermic, so it can indicate the energy fluctuations between the reactant and the product. The ΔG* characterizes the process’s spontaneity, so it can show how much energy is stored within the system, including the energy supplied from an external source. Finally, the activation entropy (ΔS*) shows a randomness of molecules during the conversion process, i.e., the unpredictability or chaos of the system. The ΔS* value can be negative or positive, with a low entropy magnitude value implying less reactivity.

Thermodynamic parameters ΔH*, ΔG*, and ΔS* are estimated using the *T_p_* values from Table 3, and kinetic parameters values (*E_a_* and *A*) from Table 4, referring to the reaction regions I, II, and III, respectively. These results are presented in Table 5.

Considering ΔH* values for all reaction regions at the various heating rates, they are all positive, indicating the strictly endothermic conditions of the conversion process. The differences between activation energies (*E_a_*) and change in activation enthalpies for regions I, II, and III are as follows: ~3.03 kJ·mol^−1^, 5.41 kJ·mol^−1^, and 7.94 kJ·mol^−1^, respectively. The smallest difference is observed for region I compared to regions II and III, which indicates that for removing retained moisture and other light volatile components, the formation of *products* is facilitated because there is a smaller potential energy barrier between the reactant and activated complex. A higher difference between ΔH* and *E_a_* for region II, and especially for region III, indicates a tendency towards higher energy consumption by the system towards higher temperature zones. Observing the influence of the heating rate (*β*) on the values of all thermodynamic quantities for separate regions, there is no significant influence of the heating rate, because there is no substantial change in them. Changes exist laterally as the temperature *T_p_* increases (Table 5). As for ΔG*, which represents a measure of the total energy increase during the reaction in a thermal conversion process, for all regions, these values are positive and increase with the transition from I → III at all *β*’s (Table 5). These results clearly indicate the non-spontaneity of the reactions, which means that reactions demand more and more input energy in order to take place (non-spontaneous). The lower values of ΔG* at all *β*’s for region I indicate that the product formation is more favorable, and less activation energy is required during the thermal conversion. The most demanding in terms of energy is region III, which draws a lot of energy for the formation of products (tending to form activation complexes with high energy) (ΔG* = +275.89 kJ·mol^−1^ (Table 5)); it requires much more energy input to complete the entire process. Considering the values of activation entropy (ΔS*), there are certain differences in the signs of the thermodynamic quantity that change across the reaction regions. Namely, for region I at all *β*’s, the negative ΔS* values exist (~−116.64 J·mol^−1^·K^−1^, Table 5), which indicates the product disorder within the CBA-TB sample, which is realized through bond separation below an initial reactant. The low ΔS* indicates that the product was near its thermodynamic equilibrium state, and the investigated material shows a low reactivity. In contrast, the high ΔS* means the high reactivity and the less time consumed to form the activated complex. So, the larger ΔS* refers to the reaction substance that is out of the thermodynamic equilibrium, suggesting the system response that reacts quickly in the current scenario, thus increasing the reactivity. A higher positive value of ΔS* for regions II and III (the largest positive values are reached for region II at all *β*’s (Table 5)) demonstrates that the product disorder due to the bond dissociation was *greater* than that of original reactants. So, these higher ΔS* for regions II and III suggest more disorder caused by the thermal disruption, whereby negative ΔS* values can only occur in the small portion of a system, such as a region I (considering the large values of ΔS* for regions II and III, the equilibrium has already been exceeded and reactivity of the system is high) (Table 5 and Figure 2). In summary, considering region I, the *negative* ΔS* values imply that a thermally stable product is produced and the thermodynamic equilibrium is established, whereas the *positive* ΔS* values for regions II and III (Table 5) suggest that the disorder degree of decomposition products is larger than that of initial reactants.

This is particularly prominent for the reaction region II (Figure 2), where the large value of ΔS* occurs (~467.30 J·mol^−1^·K^−1^ (Table 5)), indicating that the system has a greater disorder in the middle sector of the process (temperatures between 300 °C and 500 °C), so the thermal decomposition reaction of CBA-TB has difficulty achieving the thermodynamic equilibrium [71]. Generally, based on the obtained thermodynamic calculations, the CBA-TB sample is characterized by the dissociation reactions, which are endothermic with *positive* activation entropy changes, where the spontaneity is achieved at high reaction temperatures (*viz*. at high temperatures, the sufficient quantity of the heat is available for the system to form the active complex, and the chemical reaction is then spontaneous).

### 4.5. Results of a Detailed Kinetic Analysis of the CBA-TB Thermal Conversion Process

#### 4.5.1. Model-Free Kinetics

Since that model-free (isoconversional) methods represent “*modelless methods*”, this means that do not involve the reaction mechanism function at the constant conversion rate, where reaction rate is only the function of the temperature. Therefore, the activation energy can be established from the temperature dependence of isoconversional rates of the process, without previously determining the reaction model. These methods are useful in the case, when an underlying reaction mechanism of the process under investigation is unknown or complex. Results of this analysis may be desirable in a complementary study that would include the use of the combination of model-free and modelistic methods for examining the complex heterogeneous thermally activated processes.

**Figure 4:**
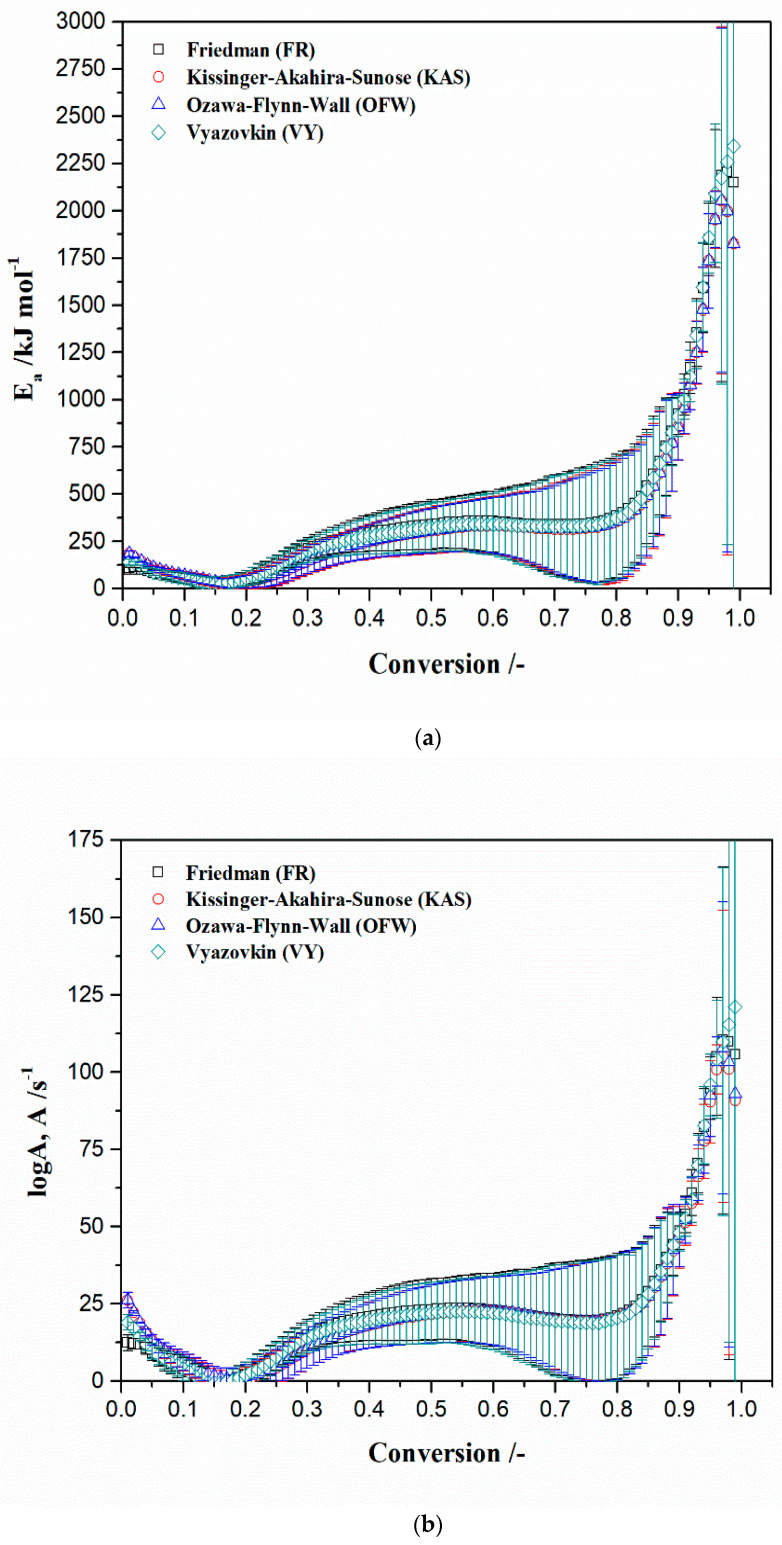
Variation of activation energy (*E_a_*) (**a**) and the logarithm of the pre-exponential factor (*logA*) (**b**) with conversion (α), calculated by means of FR, KAS, OFW, and VY model-free methods for non-isothermal decomposition process of CBA-TB.

Figure 4a,b show isoconversional dependency on activation energies (*E_a_*) and logarithm of pre-exponential factors (log*A*), obtained by Friedman (FR), Kissinger–Akahira–Sunose (KAS), Ozawa–Flynn–Wall (OFW), and Vyazovkin (VY) isoconversional (model-free) methods (Supplementary Materials), respectively, for considered decomposition process. Change of the kinetic parameters takes place with a conversion step of Δ(α) = 0.01 (α = 0.01, 0.02, …, 0.99).

From the obtained isoconversional dependencies of kinetic parameters (with respect to all applied methods), there is a very strong variation of *E_a_* and log*A* throughout the entire process progression, indicating complex multi-step reaction behavior. From the observed plots, there is an appearance of local minimum and maximum of kinetic parameters changeability with the conversion. They can be seen in the low conversion range, then in the middle, and then, in a higher conversion area (Figure 4a,b). Namely, this results in a wide range of variability in activation energies from an extreme low, then moderate, to an extreme high, suggesting different types of reaction models (mechanisms), characterized by different heights of energy barriers for starting the given reactions. As the temperature rises with the conversion advancement, the reaction rate then increases but because it causes that in a complex process, the reactions involved enthalpy shows intense changes, following the *E_a_* trends, as shown in Figure 4a. All *E_a_*-conversion profiles are characterized by an increasing trend of *E_a_* values in a wide range of conversion values, suggesting the existence of a process that involves parallel reactions but which type of reaction is present here is difficult to determine right now. This especially referred whether some of them are consecutive or single-step reactions.

This behavior clearly indicates the previous statement, that the decomposition process is relatively complicated and cannot be employed in just one reaction-step, while in the meantime, the activation energy value during the entire decomposition process, mostly exceeds the value of 150 kJ mol^−1^. Therefore, the higher activation energy means that decomposition reactions are more sensitive to the temperature, *T*. From the kinetic standpoints, in the specific case, the *E_a_* value may give an idea about thermal decomposition reaction of relative bond strengths, within the molecules studied. Factors which can affect the activation energy are the following: the temperature, concentration, and/or pressure (when the rate of reaction increases, it provides flow characteristics of bottom ash which was high, so this means that the *E_a_* becomes less), the physical state of bottom ash, the composition of bottom ash, the percentage of CaO in the bottom ash, and the basicity of bottom ash (associated with percentage attendance of CaO, where for CaO/SiO_2_ > 2, the bottom ash is considered to have high basicity). Since our sample does not show high basicity (CaO/SiO_2_ << 2) in the actual case, it cannot be expected that silicon cations form such complexes, which would move the *E_a_* value away from an extreme one. However, we can notice that very high values of *E_a_* (~1354.9 kJ mol^−1^, at the conversion of 0.93, considering FR’s method, in Figure 4a) exist, but this is not unusual, since that significant presence of MgO (Table 1) abruptly increases *E_a_* value. For an example, if we have a content of magnesia in the bottom ash of MgO = 11.2%, the *E_a_* reaches a value of even *E_a_* = 5758.5 kJ mol^−1^ [72]. Also, with an increasing of the ratio C (the unburned carbon)/S, the *E_a_* value may show a very significant increase [72]. Considering the results obtained from the standard method, the kinetic parameters estimated for the region I correspond to the values of kinetic parameters from model-free approach, at the lower conversion values (α = 0.08/0.09, Figure 4), while the *E_a_* and *A* values estimated for the regions II and III (Table 4), corresponds to the model-free kinetic parameters, at higher conversion values (II—α = 0.85 and III—α = 0.80; Figure 4). The significant differences in the kinetic parameter values for regions II and III compared to region I indicate on the strong kinetic complexity of the process, with the variety of intrinsic kinetic parameters and diversity of the reaction mechanisms. These results are in good agreement with those derived on the basis of the thermodynamic analysis in the previous sub-section (Table 5).

As a confirmation of the above facts, the existence of the activation energy distribution (AED) was found, as proof of the complexity of the investigated conversion process. This means that within the examined system, there are particles characterized by different reactivity, and they are related to the continuous distribution of kinetic parameter values for the reactions that take place as surface or bulk reactions. The existence of this distribution is a consequence of serious variation of kinetic parameters as shown in Figure 4 but in its interpretation; however, it can exist in the light of intrinsic kinetic explanations with the allocation of a specific thermal conversion stage. Among these distributions, the most frequently ones are Gaussian, Weibull, and Gamma distributions [73]. The distribution of *E_a_* values satisfies the relation as: *d*α = *Φ*(*E_a_*)*_exp_*·*dE_a_*, which reflects the probability (the experimental distribution curve, *Φ*(*E_a_*)*_exp_*) that the particles within the bottom ash have the activation energy values in the range between *E_a_* and *E_a_* + Δ*E_a_* as $\int_{E_{a}}^{E_{a}+∆E_{a}}{\Phi \left(E_{a}\right)}_{exp}dE_{a}$, whereby the distribution obeys to the normalization condition, as: $\int_{-\infty }^{\infty }\Phi {\left(E_{a}\right)}_{exp}dE_{a}=1$. The experimental density distribution function (*ddf*) can be obtained by differentiation of α(*E_a_*) curve with respect to *E_a_*, such as $\Phi {\left(E_{a}\right)}_{exp}=\frac{d\left(\alpha \left(E_{a}\right)\right)}{d\left(E_{a}\right)}$, where the term α(*E_a_*) represents experimentally derived α = α(*E_a_*) plot. After carrying out the procedure described here, it was found that the experimental distribution of the activation energies (*Φ*(*E_a_*)*_exp_*) can be described by the LogNormal distribution function in a form:

(1) $$\Phi \left(E_{a}\right)=\Phi_{o}+\frac{A^{*}}{\sqrt{2\pi \;}\sigma E_{a}}\cdot e^{-\frac{{\left[ln\left(\frac{E_{a}}{E_{a,c}}\right)\right]}^{2}}{2\sigma^{2}}},$$

where *Φ_o_* is the onset of the distribution [mol (kJ)^−1^], A* is the area of distribution function (toward to *x*-axis expressed in kJ mol^−1^), *σ* is the standard deviation (kJ mol^−1^), while *E_a_*_,*c*_ represents the central distribution value (kJ mol^−1^), and for LogNormal distribution this is equal to *median*. Other important characteristics of this distribution are given by the following equations (Equations (2) and (3)):

(2) $$Mean:\;E_{a,c}\cdot e^{\frac{1}{2}\cdot \sigma^{2}},$$

and

(3) $$Variance:\;E_{a,c}^{2}\cdot e^{\sigma^{2}}\cdot \left(e^{\sigma^{2}}-1\right),$$

whereby the variance represents the probability-weighted average of the squared deviation from the mean value. Another indicative feature of the considered distribution represents the skewness, where the skewness is a measure of the asymmetry of the distribution, and it is represented by Equation (4):

(4) $$Skewness=\gamma =(e^{\sigma^{2}}+2)\cdot \sqrt{e^{\sigma^{2}}-1}.$$

**Figure 5:**
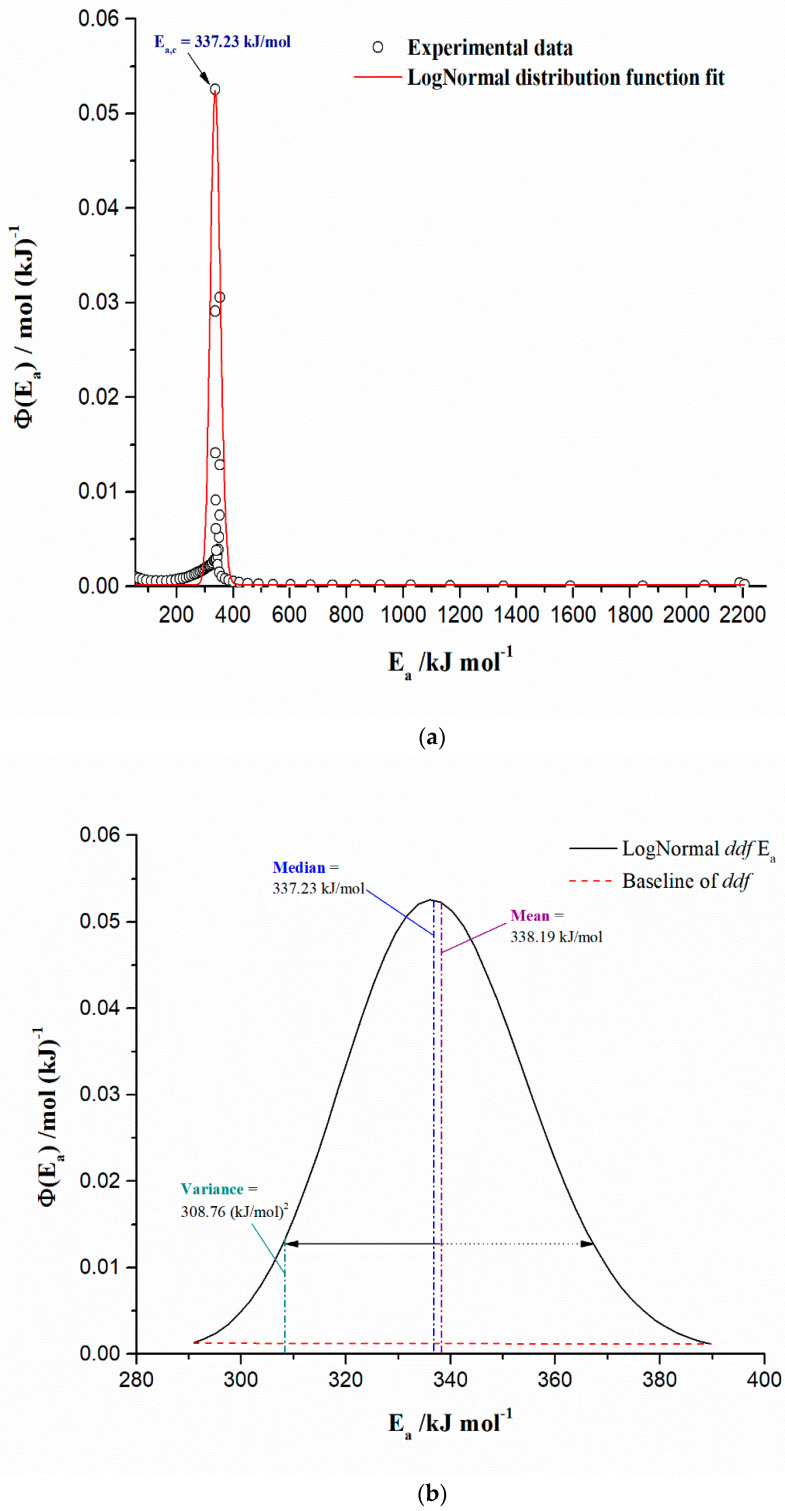
(**a**) Experimental *ddf* (the full open circles) and the LogNormal *ddf* fit (Equation (1): solid red line curve) with the indicated *E_a_*_,*c*_ valu, and (**b**) Extracted LogNormal distribution function without short—and long–tails, with the main part of the distribution (enlarged), where the central peak is found (the values of median, mean, and variance were compared) (baseline of *ddf* is represented by the dashed red-colored line).

Figure 5a shows the form of the experimental distribution of the activation energies (*E_a_*’s) (basically obtained from α = α(*E_a_*) plot by the Friedman’s model-free (isoconversional) method, but similar curves were obtained by the other methods as well) (*symbol*: the full open circles) and fitted LogNormal distribution function to the experimental data (*symbol*: solid red line curve). The LogNormal density distribution function (*ddf*) parameters are listed in Table 6.

It can be observed from the results presented in Figure 5a that the estimated distribution is characterized by many smaller *E_a_* values with a high “density” concentrated around the distribution peak and “just a few” large *E_a_* values, and these values characterize the long right–tail of the current distribution. In that sense, extremely high *E_a_*s are actually overrated. The obtained distribution is positively (+) skewed and is right-skewed distribution (*γ* = 0.156 > 0), characterized by the longer right–side tail of its peak (the long tail on its right side—Figure 5a). Therefore, the distribution is primarily characterized by the lower values of activation energies distributed symmetrically around the peak, which can be seen from Figure 5b, while in the case of the obtained distribution, it is the *mean* (338.19 kJ/mol) > *median* (337.23 kJ/mol). Both mean and median values correspond to conversion values at about α ~68% and α ~71%, respectively. These points coincide with the part of the conversion process of CBA-TB, where the “shoulder” reaction profiles occur (about 450 °C—Figure 2). These results indicate that most particles within the CBA-TB sample have such reactivity that is characterized by reduced *E_a_* values in a narrower *E_a_* interval (Figure 5b), which can participate in the parallel reaction sequence. It should be pointed out that upon reaching a temperature of *T* ~450 °C, the chromium in the tested sample can participate as the hexavalent chromium catalyst after thermal activation for temperatures up to 450 °C. In the considered case, it may happen an anchoring process of Cr(VI) through the esterification, where the silica surface hydroxyl groups are consumed as chromium and is attached to the surface by oxygen linkages [74]. Namely, the ultimate chromium/silica interactions depend on several factors, as they are textural properties of the “support”, chromium loading, the temperature, and the present concentration of oxygen in the reaction system [75].

Furthermore, however, since *σ* has a lower value, with higher *E_a_*_,*c*_ (Table 6), it is clearly seen that these results indicate that *most particles* have *higher* activation energies because of their narrower spread in distribution (Figure 5a). This requires temperature intensification of the process by converting the process into reactions demanding high temperatures to take place. The exact identification of such reactions in this case is impossible to determine, and therefore, it is necessary to apply further kinetic calculations for describing multi-step chemical reactions via the model-based analysis. Therefore, to obtain more detailed kinetic information about the investigated conversion process, a model-based approach must be applied (see the later results).

For the considered thermal decomposition process, all the applied isoconversional (model-free) methods were compared in terms of their fit to the measured TG-data (by the statistical parameters evaluation), and the results are summarized in Table 7 (more details about the statistical fitting parameters used here can be seen elsewhere [76]).

**Figure 6:**
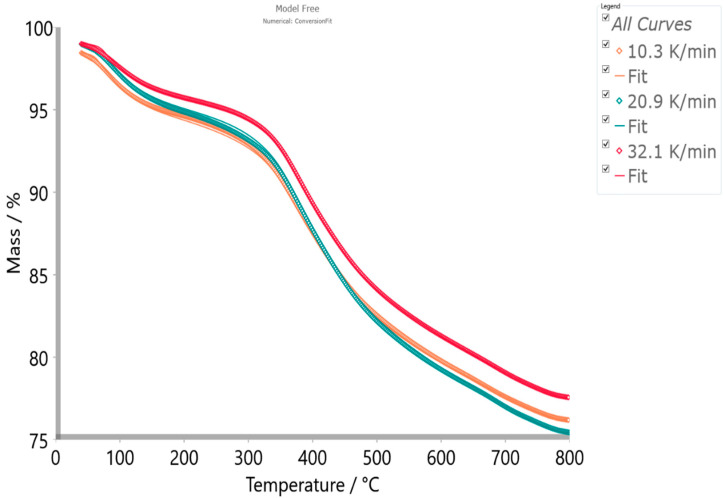
Conversion fit optimization of CBA-TB thermal decomposition process through TG-signals, using modified Friedman’s model data (*colored symbols*: experimental TG-data points; *colored solid lines*: optimized results—calculated (fit) TG-curves (Supplementary Materials, Equations (S8)–(S9)) (NETZSCH Kinetics Neo software image extracted (Product version 2.7.3.15, Build date 6 November 2024))).

The best fit was achieved using the Friedman and numerical models/methods, with an excellent fitting value of R^2^ = 0.99983, while the Vyazovkin model gives a slightly lower quality of the fit, with R^2^ = 0.99977. However, if we want to achieve a reliable calculation for the considered reaction system, then the value of R^2^ should be around R^2^ = 0.999, which provides decent predictions. Therefore, Friedman’s model was chosen among investigated models, since it provided the best fit values overall (Figure 6) (*viz*., the excellent quality of the fit shows the numerical model, since it represents the modified Friedman’s method (Section S.I., Supplementary Materials)).

##### Kinetic Compensation Effect (KCE) from Isoconversional (Model-Free) Approach

The pre-exponential factor at each conversion can be calculated based on the computed activation energy from model-free methods in advance (Supplementary Materials). It is because of the strong linear relationship between *logA* and *E_a_*, which is defined as kinetic compensation effect (KCE) that can be expressed by the relation *logA* = *a* + *b*·*E_a_*, where *a* = *log*(*k_iso_*), and *b* = 1/*R*·*T_iso_* → *T_iso_* = 1/*R*·*b*, while *a* and *b* represent constants depending on the controlled heating rate [77]. The quantities *k_iso_* and *T_iso_*, separately, represent the artificial isokinetic rate constant (s^−1^) and the artificial isokinetic temperature (°C), respectively.

**Figure 7:**
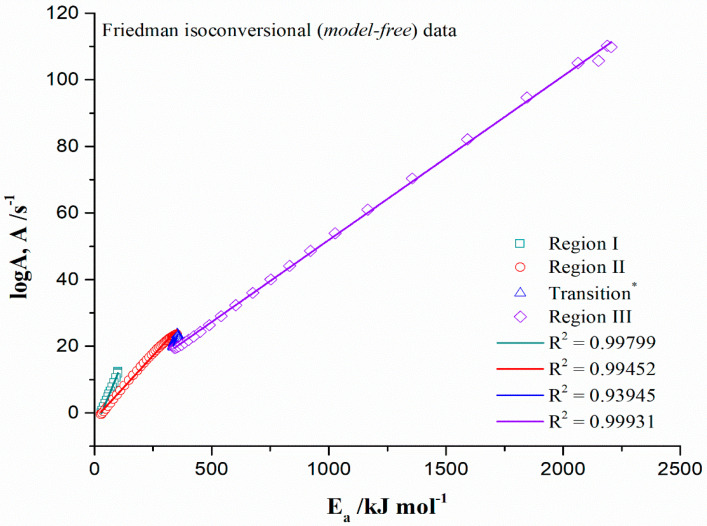
Compensatory behavior of the kinetic parameters, *logA* and *E_a_*, expressed through the KCE relationships as *logA*_(conversion (α))_ = *a* + *b*·*E_a_*_(conversion (α))_ for the thermal decomposition process of CBA-TB sample, with indicated Adj. R-Square (R^2^) values for each KCE branch (Region I: Δα = 0.01–014; Region II: Δα = 0.15–0.54; Transition*: Δα = 0.55–0.73; Region III: Δα = 0.74–0.99).

Figure 7 shows the corresponding *logA*—*E_a_* linear relationships identified from Friedman’s isoconversional (model-free) data (Figure 4a,b), which are divided into the appropriate reaction regions (KCE branches), such as Region I, Region II, Transition*, and Region III, respectively. Every compensation branch is characterized by a corresponding degree of linear correlation, suggesting a different effect of the reaction temperature on the kinetic parameters (*E_a_* and *logA*), and therefore, both, *E_a_* and *A* are strongly interconnected.

Figure 7 indicates a satisfactory linear fit for all reaction zones with a high regression coefficient (R^2^ > 0.99350), except for the Transition* area, encompassing the conversion range of Δα = 0.55–0.73 (R^2^ = 0.93945), which demonstrates a poorer approximation with *A*-values, calculated with a linear equation (the greater amplitude of errors in the specified conversion area—Figure 4a,b).

This may be explained by the fact that with the increase in the heating of the system toward the higher temperatures, the reaction rate in that part of the process decreases, expressed over a small value of *k_iso_*, indicating the reaction deceleratory character. For other regions shown in Figure 7, especially for Region III, there are strong linear correlations between *logA* and *E_a_*, indicating the mighty compensatory law between obtained kinetic parameters (there is the KCE phenomenon, which is completely real) as a consequence of the form of the Arrhenius expression.

Table 8 lists the constants *a* and *b*, and the values of the artificial isokinetic rate constant (*k_iso_*) and the artificial isokinetic temperature (*T_iso_*), for every observed KCE branch, in the case of the non-isothermal decomposition process of the CBA-TB sample. Besides the indicated values, the corresponding standard errors for these parameters are also provided.

From the results presented in Table 8, it can be observed that there are two KCEs relationships produced, which fall into two categories: (1) low gradient KCEs (Regions II and III) and (2) high gradient KCEs (Region I and Transition*). So, there is a significant difference in the magnitude of the slopes between these two categories. Surely, this is reflected in the obtained values of *T_iso_*. Namely, according to Vyazovkin et al. [78], the value of *T_iso_* can be treated as the criterion to judge the appropriateness of the studied reaction model.

So, if the value of *T_iso_* is outside of the range of the experimental decomposition temperatures, then the reaction model is not suitable or cannot be precisely determined. On the other hand, if the value of *T_iso_* is within the range of the experimental decomposition temperatures, then this indicates the proper reaction model, which can be used in the modeling or in trustworthy prediction about the type of reaction involved in a multi-step process. Considering isoconversional models applied, they lead to very high *T_iso_* values out of the scope of experimental decomposition temperatures (in the conversion ranges of Δα = 0.15–0.54 and Δα = 0.74–0.99 for Region II and Region III, respectively) (Table 8), where the behavior *T_iso_* → ∞ is valid. The observed cases correspond to the process segments where an increase of *E_a_* and *logA* values with conversion (α) exist. Namely, such behavior corresponds to the appearance of parallel reactions, which may propagate through the sequential (consecutive) type of reaction mechanism. This may appear by two successive simple reactions, where each reaction in the sequential step is characterized by different activation energy values (as *E*_1_ ≠ *E*_2_), related to the individual step. However, referring here to this complication of the process, which type of the kinetic model function (Supplementary Materials) was involved is very difficult to determine (see above discussion). Such kinetic complexity pushes the process towards very high temperatures (see the results from thermodynamic analysis), in order to break down molecularly complex systems into simpler ones and more stable reaction products, making *k_iso_* high (Table 8). Therefore, *T_iso_* represents apparent or truly artificial isokinetic temperature in the true sense of this term, and the isokinetic relationship (IKR) does not exist. For the remaining two cases, where *T_iso_* ∈ *T_exp_* (Region I and Transition* (Table 8)), there is a significantly less pronounced variation of *E_a_* and *logA* values with conversion (α), but with convex *E_a_*(α) and log*A*(α) dependencies (Figure 4). This may suggest the process with a change of the rate-limiting step in which the lower reaction temperatures dominate, i.e., the favor of lower heating rates, as well as the low values of *k_iso_*. Namely, an increase of the *E_a_* is usually associated with an increase of *A*, so the former causes the decay of the reaction rate and the latter an enhancement, whereas a decrease of the first parameter (a positive effect on the reaction rate) is very often, associated with a decrease of the second (a negative effect on the reaction rate), thus leading to the appearance of KCE. Since the linear relationship between *logA* and *E_a_* is present among various decomposition regions but with varying degrees of linear fit qualities, which means that the compensation lines are not the same (Table 8), this may suggest the existence of certain differences in structures and stability of CBA-TB active constituents, as well as the intermediate products, which were formed. Considering these results, they undoubtedly give us strong theoretical evidence for adequately choosing different types of reaction steps (and the complete reaction model reconstruction) for the later modeling of the decomposition process.

It should be emphasized that unlike the model-free methods, which have certain drawbacks, the model-based method uses a searching pattern that takes into account the selected number of isoconversional lines. It was based on the utilization of clarity and magnitude of derived error bars (indicated by standard deviation) from model-free generated kinetic parameters in the adjacent averaging within the fits cycle of the data. This is especially important if there are accelerating reactions and significant thermal effect changes (Supplementary Materials) inside the reaction system.

#### 4.5.2. Model-Based Kinetics

The obtained Friedman’s isoconversional data were used as the initial parameters searching tool to generate the reliable kinetic triplets, for a physicochemical description of the entire thermal decomposition mechanism, using the model-based approach (Section S.II., Supplementary Materials). Model-based methods involve the fitting of mass (conversion)—temperature curves by the different models, and simultaneous determination of the kinetic parameters such *E* and log*A*. This kinetic approach assumes that the process has several steps, each of them has its own kinetic equation, while the kinetic parameters of each step possess constant values. For the determination of the kinetic triplet [*E*, *A*, *f*(α)] of CBA-TB decomposition process, the various reaction models were considered (Table S1, Supplementary Materials) for the fitting of the experimental data at the different heating rates, using the multivariate non-linear regression method. Uses the model-free results, which reveal that decomposition process proceeds through a reaction pathway that consists of more than one mechanism, multivariate non-linear regression is applied on the thermo-analytical (TA) data, so the following decomposition mechanism scheme was established, as

(5) $$A\;\overset{An}{\to }\;B\;\overset{F2}{\to }\;C,$$

(6) $$D\;\overset{Fn}{\to }\;E\;\overset{R3}{\to }\;F,$$

(7) $$G\;\overset{Cnm}{\to }\;H,$$

and designated as the p:, model reaction scheme. The process scheme consists the two sequential (consecutive) reactions steps, and one single-step reaction step, described by Equations (5)–(7), respectively. In the considered reaction scheme, *A*, *D,* and *G* represent reactants, *B* and *E* are the intermediate species, while *C*, *F,* and *H* represent the products. The first sequential stage is depicted by an *n*-dimensional nucleation (Avrami–Erofeev equation) model, A*n*, *f*(α) = *n*·(1 − α)[−ln(1 − α)]^1−1/*n*^ (step: *A* → *B*) and by the second-order chemical reaction, F2, *f*(α) = (1 − α)^2^ (step: *B* → *C*). The second sequential stage is described by an *n*-th order chemical reaction (*n* ≠ 1), F*n*, *f*(α) = (1 − α)*^n^* (step: *D* → *E*) and by the phase boundary-controlled reaction (the contracting volume model, 3D), R3, *f*(α) = 3·(1 − α)^2/3^ (step: *E* → *F*). Finally, the single-step reaction (Equation (7)) is described by the *n*-th order and *m*-power reaction with the autocatalysis, *Cnm*, *f*(α) = (1 − α)*^n^*·α*^m^* (step: *G* → *H*) (Table S1, Supplementary Materials). The model-based machinery works by a reducing difference between the experimentally measured and the calculated data on the reaction rate, using the non-liner regression analysis in the case of the high kinetic complexity of studied process. Results of the model-based analysis, which concludes the best selected kinetic models, through the model scheme code p: (considering CBA-TB decomposition mechanism in A*n*F2F*n*R3C*nm* kinetic models sequence) that includes the reaction steps described by Equations (5)–(7) are presented in Table 9.

Based on the obtained mechanistic scheme, the following conclusions can be drawn:

- The first step ($A\overset{An}{\to }B$) in the sequential reaction stage (see Equation (5)) which takes place in the temperature range of Δ*T* = 40–700 °C, is attributed to the reduction reaction of the ferric oxide (hematite) into iron(II, III) oxide (known as black iron oxide–magnetite) with the release of molecular oxygen, and the simultaneous elimination of the physically adsorbed water:
  Fe_2_O_3(*s*)_ → (2/3)Fe_3_O_4(*s*)_ + (1/6)O_2(*g*)_↑. (8)

Based on the obtained Avrami dimension parameter (*n*) which amounts *n* = 0.652 (Table 9), this suggests that the magnetite nuclei were not only situated on the particle surface, but also in the inner area of the particle, i.e., the former is the surface nucleation, and the latter would suggest the bulk nucleation (bulk nucleation could be a dominant process in the reaction) [79]. Based on the obtained lower value of the activation energy for this step (*E_a_* = 24.842 kJ/mol), there is probably a removal of the total oxygen content in hematite, where the reaction is very fast (with a low energy barrier) at the beginning (lower temperature region and lower conversions, α ~0.15 and then slowed down or stopped, with the transition to the high-temperature region and towards the higher conversions.

- The second step ($B\overset{F2}{\to }C$) in the sequential process stage (see Equation (5)) which occurs in the temperature range of Δ*T* = 200–800 °C can be attributed to Fe_3_O_4_-FeO interconversion, strongly assisted by the oxygen emission from previous step (Fe_3_O_4_ is the intermediate chemical specie “*B*”), favors the production of FeO (wüstite) [80]:
  (9) $$Fe_{3}O_{4(s)}\;\overset{+the\;presence\;of\;CO}{\overbrace{\overset{\left[O\right]}{\to }}\;\;FeO_{\left(s\right)}},$$
  which represents the final reaction product. This reaction was additionally promoted by the presence of CO liberated from CBA-TB, since that sample has high affinity for the association of present carbon and high oxygen concentrations towards CO generation. So, the second-order (F2) kinetics identified for the current reaction step is related to concentrations of Fe_3_O_4_ and CO present within the reaction system, which could be a platform for oxidation-reduction (redox) reaction (where CO is a reducing agent, while Fe_3_O_4_ is an oxidizing agent) forming a significant amount of iron(II) oxide, with allocation of CO_2_ as by-product.

It should be emphasized that obtained activation energy *E_a_* = 217.334 kJ/mol (Table 9) is much higher than activation energy for one-step magnetite reduction in the presence of hydrogen (H_2_) into the metallic iron (~55 kJ/mol) [81]. The reduction potential strongly depends on the ratio of CO/CO_2_ present in the system at the higher temperatures, influencing the values of kinetic parameters. Consequently, based on the estimated value of *E_a_*, it can be concluded that observed reaction step proceeds through solid-state controlling mechanism—the reaction is controlled by nucleation model (unlike the previous reaction step *A* → *B*, which was controlled by solid-state diffusion) but not excluding an amorphous graphite-like deposits and the metallic iron phase islands. Therefore, it is interesting to note here that relatively high activation energy (=217.334 kJ/mol) is a consequence of the oxide to carbon ratio or in other words, the possible presence of the “coupled” gaseous reduction of Fe_3_O_4_ (Equation (9)) and the gasification of carbon [82], which exists in the sample. It should be noted that further conversion of FeO into the metallic iron strongly depends on the time duration and concentration of CO present in the system. However, this process can be highly promoted from a temperature of 800 °C, which is beyond the scope of this study. But obviously, the effect of temperature is present, so, in the considered temperature range, the certain content of the carbon exists, and may probably arises from Boudouard reaction [83], which decreases as the temperature increases. It can be supposed that after 700 °C, the process reaction becomes slower, as a result of the bonding and more difficult penetration of CO gas through subsequently formed layer around the particles. Based on these facts, the previous step represents the controlling step for the current consecutive reactions stage, emphasizing the diffusion phenomena.

- In addition, the first reaction step ($D\overset{Fn}{\to }E$) in another sequential stage (see Equation (6)) which takes place in the temperature range of Δ*T* = 40–200 °C exhibits strong overlapping behavior with previous sequential stage, especially with *A* → *B* reaction step, in the lower temperature zone. This step was characterized by anorthite (CaAl_2_Si_2_O_8_) *P*1–*I*1 phase transition occurring up to temperature of *T* = 200 °C [84,85]. Actual designed experimental conditions for thermal decomposition of CBA-TB specimen are sufficient for thermal disordering pathway, whereby *I*1 phase may occurs at the lower temperatures, whereabouts pressure in the system is a high (in general, *P*1–*I*1 phase transition occurs from “low pressure” into “high pressure” system behavior) [86]. This transition is associated with a re-organization of the lattice modes, accompanied with structural changes. These changes indicate on the onset of strong distortion of the aluminosilicate framework of the *I*1 phase but the duration of this distortion does not go to infinity with further increase of the pressure in a system [87]. It is interesting to note that depending on magnitude of this pressure in the system, the kinetic character of this transition will also depend. It was founded that *I*1 phase of anorthite is stable at least up to 8.8 GPa but at a higher pressure out of 8.8 GPa, specifically on 10 GPa, the transition is the first-order in its nature [87]. For the actual step, we obtained that the kinetics of the transition goes by *n*-th order kinetics, with *n* = 1.975 (Table 9), indicating that a stable anorthite *I*1 phase has been reached, where no further transformation into a new high-pressure polymorph occurs. Namely, the first-order and *n*-th-order transition kinetics can be referred to as unstable ‘*very high-pressure*’ and stable ‘*high-pressure*’ anorthite *I*1 phase formation [88], which also affects the value of activation energy (Table 9). The *P*1–*I*1 phase transition is strongly influenced by both the composition and the temperature. Therefore, the activation energy for thermal disordering strongly depends on composition and temperature, especially on the ratio of SiO_2_/CaO present in the studied sample. For our sample, this ratio amounts of SiO_2_/CaO = 4.92. As this ratio is higher, the higher the *E* value (Table 9). On the other hand, the weak crystallization tendency of anorthite is guided by the increased SiO_2_/Al_2_O_3_ ratio [89]. For the investigated sample, the indicated ratio amounts to the SiO_2_/Al_2_O_3_ = 2.04. The increase of this ratio above 2.00 causes an increase in an energy barrier for the crystallization. The SiO_2_/Al_2_O_3_ ratio slightly exceeds the above-indicated value of 2.00, which is enough to prevent the trigger for the pure crystallization process.
- The second reaction step ($E\overset{R3}{\to }F$) within the sequential stage described by Equation (6) occurs in the temperature range of Δ*T* = 200–800 °C, and this can be attributed to anorthite *I*1 phase breakdown reaction. The actual reaction includes an anorthite softening step or nucleation, where the incongruent melting product can be obtained at temperatures above *T* ~700 °C [90]. The process was governed by thermal dissolution, which starts on the grain with the most soluble surface, most likely as ‘crystallographic’ controlled. The mechanism proceeds through a phase boundary-controlled reaction (R3, contracting volume 3D, with a geometrical factor of *n* = 2/3). The geometric contraction volume (R3) model is associated with low activation energy (*E_a_* = 30.830 kJ/mol; Table 9) for the migration of tetrahedrally bonded atoms. It is possible that there is a pronounced meta-stability near the phase boundaries, which inhibits the formation of inter-growths. Since the observed kinetics does not take place according to the first-order kinetics but according to deceleratory contracting reaction mechanism (R3), there is a high probability that the transition takes place through retarding a variation of Al-Si order with an increasing temperature. As the final product, CaO·Al_2_O_3_·2SiO_2_ melts with CaO allocation are obtained [91].
- The last one, the single-step reaction ($G\overset{Cnm}{\to }H$) (see Equation (7)), which takes place in the temperature range of Δ*T* = 300–800 °C is characterized by *Cnm* model—*n*-th order and *m*-power reaction with autocatalysis (Table 9). The current reaction step has autocatalytic nature with an activation energy of 224.651 kJ/mol, with acceleratory portion order of *m* = 0.170 (the product involvements in reaction acting as the catalyst) and *n*-th order exponent of *n* = 6.392 that characterizes consumption of the reactant (Table 9). It is interesting to note that this reaction appears in the decomposition zone, where “shoulder” feature arises (see previous results). This stage can be attributed to the chromium-incorporated SiO_2_ decomposition, which is more thermally resistant than the CrO_3_ specie alone. This is probably a consequence of the highly dispersed chromium on the silica surface, resulting in the decomposition of “impregnated” catalyst by the following reaction:
  Silica/Cr(VI) → Silica/Cr(II) + O_2(*gaseous*)_↑. (10)

Namely, the recombination of two terminally bonded oxygen atoms within isolated chromate-like species, leading to evolution of the oxygen molecule, which results in Cr(II) oxo-species, according to Equation (10). Also, this reaction may be promoted (the increased yield of Cr(II) species) with forcefully reduced content of free water in the system, and with increased content of CO, which would act as the reducing agent. Consequently, Cr(II) oxo-species can act as the catalytic sites on the silica surface. It should be noted that one of their important applications is to represent an effective catalytic site in the ethylene polymerization [92,93,94]. This is an important processing cycle in the petroleum industry of converting light olefin gases into the hydrocarbons of the higher molecular weight (MW), which would be used as the gasoline blending stocks [95].

**Figure 8:**
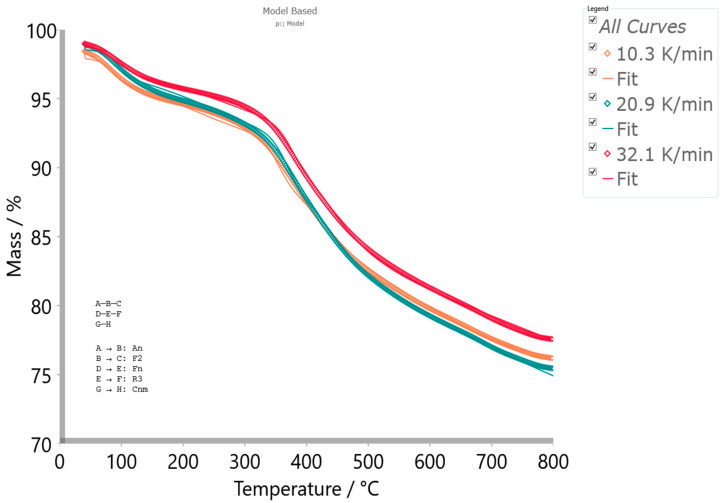
Model-based TG-signal fits to experimental data, based on the proposed p:, model scheme, at the various heating rates, for thermal decomposition of CBA-TB (*colored symbols*: experimental data points; *colored solid lines*: model-based results (R^2^ = 0.99968)) (NETZSCH Kinetics Neo software image extracted (Product version 2.7.3.15, Build date 6 November 2024)).

Based on the summarized model-based kinetic analysis results, the accuracy of these results was verified by the fitting with an experimental thermo-analytical (TA) data, so, the comparison between calculated (through the established p:, model) and experimental data, is shown in Figure 8.

From the results presented in Figure 8, it can be seen that there is a correct and satisfactory interpretation of the experimental data by the proposed mechanism scheme (p:, model scheme), where fitting quality is a slightly lower (R^2^ = 0.99968) than the optimization and Friedman’s approaches (see above). This result can be taken with great confidence, considering that for decent description of entire decomposition process, the correlation coefficient, such as R^2^, should be sufficiently high, ideally around ≈ 0.999.

**Figure 9:**
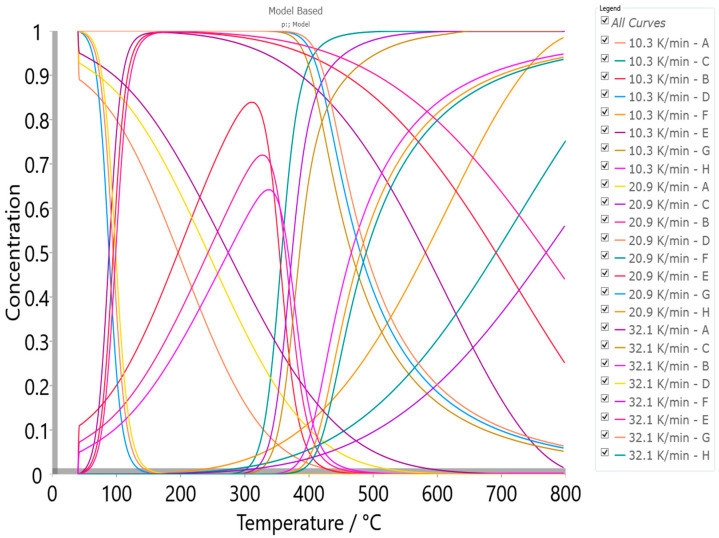
Normalized concentration species evolution with the temperature, for every reaction step in the proposed p:, model scheme at the heating rates of 10.3 K/min, 20.9 K/min and 32.1 K/min (NETZSCH Kinetics Neo software image extracted (Product version 2.7.3.15, Build date: 6 November 2024)).

**Figure 10:**
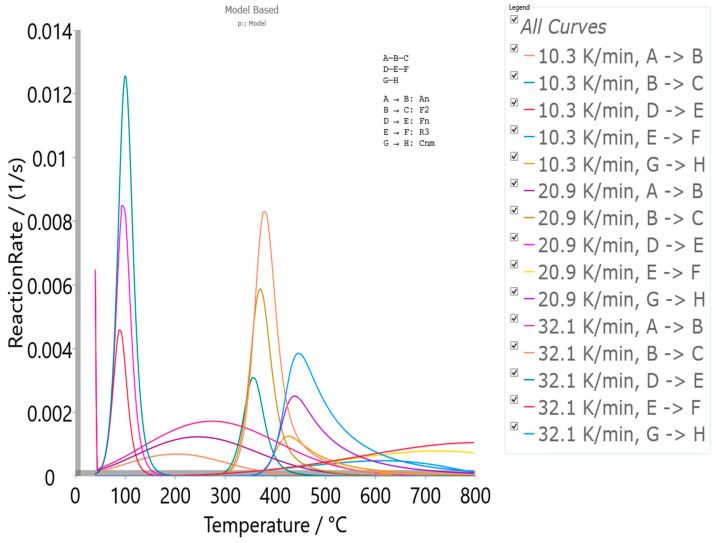
Reaction rate curves (1/s) vs. the temperature (°C) for all CBA-TB decomposition steps described by p, model scheme, which is determined using the model-based kinetic approach at the different heating rates (NETZSCH Kinetics Neo software image extracted (Product version 2.7.3.15, Build date 6 November 2024)).

Figure 9 shows the normalized concentration species evolution with the temperature, during the entire thermal decomposition process, including the reaction scheme shown by Equations (5)–(7). In addition, Figure 10 represents the reaction rate curves (1/s) against the temperature (°C) for every considered reaction step in the p:, model scheme, considering the applied set of the heating rates. Both reaction mechanism image representations encompass the active reactants, the intermediate chemical species, as well as the final products, which are described under the above items.

From the results presented in Figure 9 and Figure 10, there are full agreement of the reactions feature evolutions with the advancement of the decomposition process temperature with ones that were explained in the above Discussion Section. It can be observed that the CBA-TB decomposition process is characterized by a strong overlapping character of reactions, which makes it a kinetically complex process. The largest contribution to the overall decomposition process has a reaction that produces CaO·Al_2_O_3_·2SiO_2_ system (contribution: 30.4%), reaching its maximum rate within a high temperature zone at the highest heating rate. However, the rise in the final product concentration for considered step is dependent on the heating rate, while the lower heating rates are more suitable. The second reaction in terms of the contribution to the overall process represents the autocatalytic step described by *Cnm* model (contribution: 23.6%). There is acceleratory (sigmoid) uprising of the concentration of the product of the reaction, which starts at about *T* ≈ 360 °C, considering all applied heating rates (Figure 9). This reaction step is characterized by asymmetrical reaction rate curves, with pronounced right–tails toward the higher process temperatures (Figure 10). The strength of the autocatalytic reaction can be estimated from the ratio of autocatalysis factor, *K_cat_* (= 8.701 (Table 9)) and the temperature T* (which represents the average temperature at the maximum reaction rate of the interest at all heating rates applied, considering Figure 10, this temperature amounts to T* = 420 °C (= 693.15 K)). Based on the implemented ratio *K_cat_*/T*, the final result was obtained, and it amounts to *K_cat_*/T* = 1.25 × 10^−2^. This value matches the autocatalytic strength classification defined as *less strong*, between 1 × 10^−2^ and 1 × 10^−3^, since the strong autocatalysis behavior is distributed between 0 and 1 × 10^−3^ [96]. Considering this fact, an asymmetrical reaction rate behavior is the consequence of combination of *n*-th order and autocatalysis reaction (Table 9), which exhibits an accelerating performance. In the considered case, there will be a certain induction period, and after that, the reaction’s acceleration becomes more significant than the *n*-th order but not as dramatic as a pure autocatalytic reaction (where *n* > *m*) (then, the full symmetry of the reaction rates at different heating rates would be achieved—Figure 10). Therefore, the proposed decomposition mechanism realistically reflects all possible transformations that occur inside the reaction system, revealing the specific physicochemical characteristics of the investigated precursor material.

### 4.6. Thermal Safety Analysis

The actual analysis was introduced in order to simulate the process that would take place in the differential scanning calorimeter—DSC or in accelerating rate calorimeter (ARC) (Supplementary Materials). However, it is known that the results obtained from these experiments cannot be directly applied at the large-scale industrial enforcements. Distinction is reflected in a fact that in the industrial production we deal with the heat accumulation in a large amount of the compound, while the DSC tests were carried out with milligram-scale samples. This is important how to handle this type of the material for industrial applications, in terms of its safety to preventing an eventual occurrence of runaway reaction (Supplementary Materials). So, the kinetic model can be employed for the prediction and simulation to evaluate the thermal safety because kinetics of any reaction does not depend on the mass of the sample [97].

Based on the kinetic model and kinetic parameters of decomposition process determined in measurements and carried out in the pure adiabatic conditions (in an absence of container) (Supplementary Materials), applying an appropriate kinetics simulation by Kinetics Neo software, the thermal safety of CBA-TB was predicted. Thermal safety of CBA-TB sample was implemented through determination of adiabatic time to the maximum rate (TMR). TMR is determined from self-heating rate for the reaction course as the time instant that corresponds to the maximal value of d*T*/d*t* (Supplementary Materials). Conditions for the adiabatic simulation based on the estimated Friedman (FR), Numerical, and p:, model results from the *T_D24_* was derived, and they are listed in Table 10.

**Figure 11:**
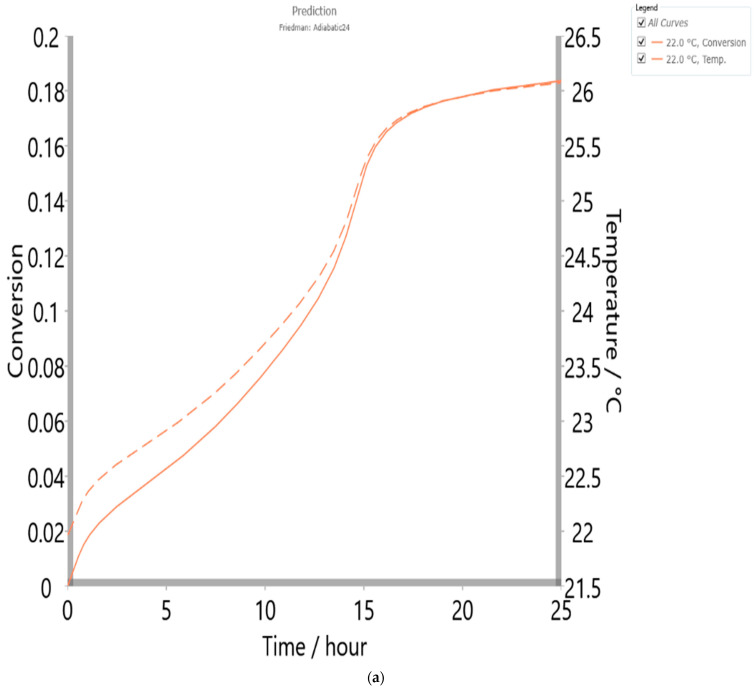

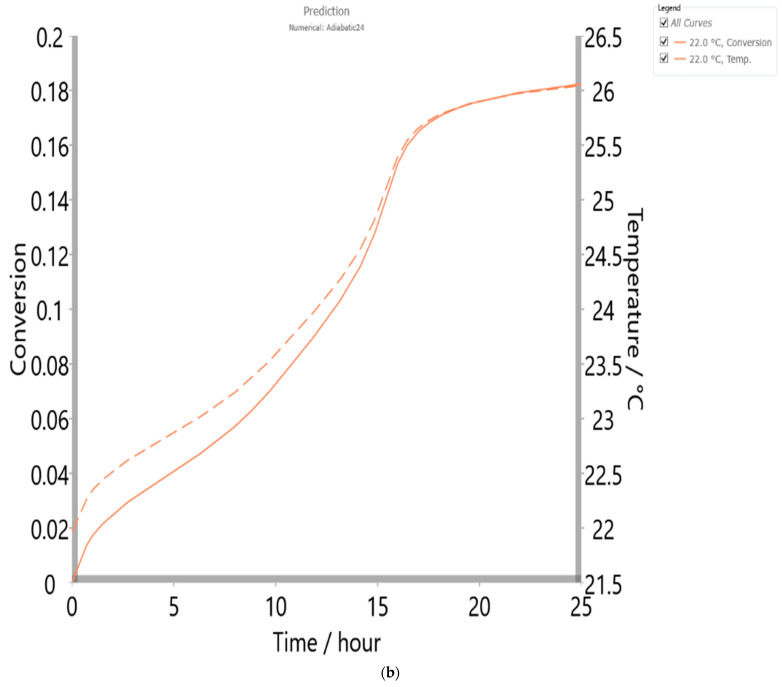

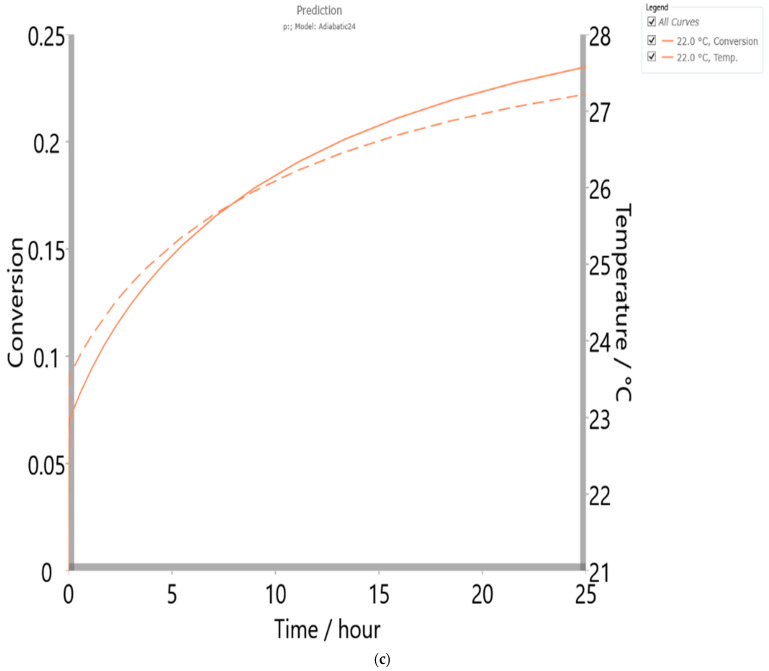
α = α(*t*) adiabatic conversion curves for CBA-TB thermal decomposition after 24 h, obtained for: (**a**) Friedman, (**b**) Numerical, and (**c**) p:, models, respectively (*full colored line*—calorimeter measurement/experimental signal; *dash colored line*—simulated signal to time to the maximum reaction rate, e.g., 24 h) (NETZSCH Kinetics Neo software images extracted (Product version 2.7.3.15, Build date 6 November 2024)).

For a given set of parameters used in the simulation (Table 10), the corresponding adiabatic α = α(*t*) curves for the occurrence of thermal decomposition after 24 h, including Friedman, Numerical, and p:, models are presented in Figure 11a–c, respectively. Therefore, once the kinetic parameters were determined with model-free and model-based methods, the thermal phenomenon for the considered sample under adiabatic conditions continued, can be successfully simulated. From the obtained results, it can be observed that in the case of model-free approach (Friedman and numerical), both curves exhibit the same shape, joining together within the high TMR and at *T_D24_* (Figure 11a,b). The shape of α = α(*t*) curve for the Friedman’s and numerical models is typical for low thermal inertia (a strict adiabatic conditions). On the other hand, the thermal curves obtained for p:, model change their shape in a profiled deceleratory form. This corresponds to the situation where the thermal inertia increases (the *ϕ* factor is above the unity) and where the conversion value expansion occurs by going to the *T_D24_* point (Figure 11c).

Accordingly, the slightly different values of *T_D24_* were obtained, such as: *T_D24_* = 218 °C for cases in Figure 11a,b and *T_D24_* = 274 °C for the case in Figure 11c, respectively. In the latter case, we have a real influence of the melting phase of anorthite on the entire decomposition process under a given condition. This essential difference cannot be seen from the model-free models, because they suffer from not recognizing an exact kinetic mechanism (involving a finite number of elementary reaction steps) of a given transformation. On the thermal safety analysis of CBA-TB sample, a formed incongruent melting product has a significant impact, so that, the model-based results have an incalculable advantage in this matter compared to model-free results when an investigated process is simulated under the above-stated conditions.

Based on the generated simulation conversion–time (α–*t*) curves, there is a different shapes of the observed plots depending on the model used, which causes the change in the *ϕ* factor (Figure 11a,b): the low thermal inertia (*ϕ* < 1) (low *ϕ* indicates that in this case, the material does not retain the heat well, meaning it is heat up and cool down rapidly; however, the ‘advantage’ of low *ϕ* is that there will be a little thermal dilution) and Figure 11c: the high thermal inertia (*ϕ* > 1) (increase of *ϕ* indicates that the measured initial decomposition temperature moves to higher values and calculated kinetic parameters, such as *E* and *A*, will have realistically the higher values). Therefore, this change in the *ϕ* factor, considering both kinetic approaches, has an effect on the *T_D24_*. Namely, the material with high *ϕ* has a higher heat capacity and can be used in the heat storage systems to store excess heat and release it when needed.

So it is an obvious, that in the considered cases, the model-free models more closely match the measurements in the absence of container (all the heat released is used to heating the sample only), while the model-based (p:,) model corresponds mostly to the measurements in the presence of container (we have the heating of the sample with container), and this causes *T_D24_* (non-container) = 218 °C < *T_D24_* (container) = 274 °C. This differentiation is probably because starting temperatures are strongly influenced the time to the rate of the temperature evolution under adiabatic conditions and the *T_D24_* value as well (see above considerations). It can be observed that *T_D24_* (non-container) is lower for 56 °C than *T_D24_* (container), affecting that the onset temperature of CBA-TB sample measured in non-container vessel was 20 °C lower than that for the CBA-TB sample tested in the container vessel, which decreased the safety of the sample. This can be observed in Figure 11c, where the calorimeter–experimental curve (full colored line) overcomes the simulated curve (dash colored line) at *T_D24_*, where its rise begins even at T*_D7.5_*. So, the kinetic triplets encompassing the reaction scheme presented by the p:, model are more appropriate for non-adiabatic conditions (where *ϕ* (*phi*) > 1 in the ARC experiments). Namely, considering the low initial temperature, the calorimeter test starts for totally not decomposed sample (α = 0), which is very difficult to achieve from the experimental point of view (considering *ϕ* = 1), while some minor reaction progress (α) occurs, during an initial period (α ~0.02), when the sample is still in the heat–wait–search modes. Consequently, when the detection limit of ARC apparatus is reached, the reaction progress (α) is no longer equal to zero, but already α > 0 (Figure 11). Having the kinetic description of the reaction, considering both model-free models (Figure 11a,b), the curves follow the same trend for the estimated reaction progress, α, after 16 h that amounts to about α = 0.17 (~17%), where the remaining TMR up to TMR = 24 h is exactly 8 h. Presented results show that the good fit of simulated and experimental results for model-free models can be additionally applied for the verification of calculated kinetic parameters. In contrast, model-based kinetic parameters are more adopted for “pseudo-adiabatic” conditions (Figure 11c). At the constant temperature, it can be seen from Figure 11a,b that α after 1 day amounts only to α = 0.18 (~18%). The decrease in *ϕ* (*phi*) value significantly changes the time required for the total decomposition. As presented in this figure, the total decomposition under fully adiabatic conditions occurs after 1 day (24 h). However, looking at the case presented in Figure 11c, the total decomposition takes place after 1 day but achieves a much higher conversion, α (about α = 0.22 (~22%)) and the higher constant temperature, directly, thereby changing the *ϕ* (*phi*) factor into *ϕ* > 1. This represents the main cause for elevated *T_D24_* for the kinetic data simulations, obtained from the model-based kinetic analysis. So, the model-based kinetic parameters more reflects the real (*apropos* ‘*observed*’) situation in terms of thermal safety analysis for considered sample, where uncontrolled reaction progress depends not only on experimental setting (i.e., the choice of initial temperature in an adiabatic experiments) but also on the kinetics of thermal conversion process [98]. Depending on the kind of rate-controlling step (the reaction step: *E* → *F*), this affects the preliminary α-value on the TMR which may be different. Considering determined *T_D24_* values which are very high, this result confirms a high thermal stability of the tested sample.

This testing showed that with the help of *T_D24_* analysis, it is proven that if the CBA-TB was used in the chemical industry in highly energetic synthesis reactions, the sample would not lead to a runaway reaction causing the hazard of an explosion due to very intense heat generation. However, this applies to the conditions presented in Table 10. For future work, a whole range of different scenarios different than the one shown in the mentioned table must be taken, thus expanding the range of conditions prevailing in different industrial plants that would be considered for specific cases.

## 5. Conclusions

By using the thermal analysis techniques, thermal stability, and non-isothermal decomposition kinetics of the coal bottom ash (CBA-TB) were investigated. The chemical, structural, and radiological characterization of the CBA-TB sample was carried out, using WD-XRF, ICP-MS, XRD, Gamma spectrometry, and gross alpha/gross beta activity techniques. Based on the obtained XRD data, it was found that the CBA-TB contains these compounds by percentage in a following order: anorthite (CaAl_2_Si_2_O_8_) > muscovite > SiO_2_ > MgO > Fe_2_O_3_. It was established that the thermal decomposition of CBA-TB exhibits a complex multi-step kinetic character, which was identified by model-free (isoconversional) analysis and confirmed by means of the estimated thermodynamic parameters values (changes in the activation enthalpy (ΔH*), activation entropy (ΔS*), and Gibbs free energy of activation (ΔG*)), and the subsequently with a model-based kinetic approach. The process proceeds through strong reactions overlapping behavior in different temperature regions. Model-free kinetics analysis showed that the process is distinguished by the higher activation energy, indicating that decomposition reactions are more sensitive to temperature variation (higher temperatures are required for these reactions to start—these results were confirmed by the derived activation energy distribution function). As a consequence of the above, the kinetic compensation effect (KCE) was also observed. Using rigorous statistical tests about the data quality fitting, it was shown that the best fit is achieved with the Friedman’s and numerical methods, without reaction model consideration. For further kinetic analysis, the results obtained from Friedman’s method were included as the starting parameters for the search of the optimal kinetic triplets of individual reaction steps within the model-based kinetic analysis.

The model-based analysis was revealed that the thermal decomposition of CBA-TB takes place through two sequential (consecutive) reaction steps and one single-step reaction, described by variety of the reaction models, including the nucleation/growth, phase boundary-controlled reaction, second/*n*-th order chemical reactions, and the autocatalytic models. The nucleation and growth mechanisms characterize the reduction reaction of ferric oxide (hematite) into magnetite with the release of molecular oxygen. It was established that bulk nucleation represents a dominant process in the actual transformation. The second-order reaction mechanism was attached to thermal transformation of magnetite into wüstite via Fe_3_O_4_-FeO interconversion. It was found that the *n*-th order reaction mechanism characterizes anorthite *P*1–*I*1 phase transition, which is strongly governed by a magnitude of the reaction system pressure (*P*). In addition, the deceleratory contracting reaction mechanism (R3) was responsible for the anorthite *I*1 phase breakdown reaction pathway. It was concluded that this step includes anorthite softening step or nucleation, where the incongruent melting product is obtained (CaO·Al_2_O_3_·2SiO_2_ melts) (it was identified that the actual step represents a rate-controlling step). Finally, the autocatalytic reaction step (described by the *Cnm* model) was attributed to the chromium-incorporated SiO_2_ catalyst decomposition, leading to formation of Cr(II) oxo-species product.

The thermal safety analysis performed under the strictly adiabatic conditions showed that obtained model-free and model-based results converge more towards to pure adiabatic and “pseudo-adiabatic” conditions (which are reflected through the different thermal inertia values), giving *T_D24_* values with displacement effect, such as *T_D24_* (non-container) = 218 °C < *T_D24_* (container) = 274 °C. The changing in *ϕ* (*phi*) factor and significant change in the degree of reaction progresses, comparing the experimental calorimeter and simulated signals have a strong impact on the *T_D24_* increasing. It was concluded that the model-based kinetic parameters reflect real situation in terms of thermal safety analysis of the investigated material. It was deduced that kind of the rate-controlling step has an impact in a preliminary reaction progress on the time to the maximum rate (TMR) in adiabatic conditions, and this influence can be different, considering both model-free and model-based approaches. In general, considering very high *T_D24_* values, the investigated sample (CBA-TB) exhibits the high thermal stability, pointing to the low thermal risks.

Presented research gives an opportunity to see advantages of the proposed procedure in the thermal conversion of coal bottom ash at temperatures below 1000 °C in order to achieve a high efficiency in its thermally activated recovering into the valuable products.

## Figures and Tables

**Table 1:** Minerals composition and heavy metals (HMs) content in the CBA-TB sample (values in parentheses represent the values reported from the literature).

Content of Basic Component (%)		Heavy Metals (mg·kg^−1^)	
SiO_2_	46.84 (52.2) ^a^, (52.2) ^b^, (52.4) ^c^, (48.2) ^d^	Pb	10.6
Al_2_O_3_	22.99 (27.5) ^a^, (27.5) ^b^, (27.5) ^c^, (25.55) ^d^	Cd	0.0
TiO_2_	1.08 (1.53) ^a^, (0.97) ^c^, (1.5) ^d^	Zn	13.4
Fe_2_O_3_	9.40 (6.0) ^a^, (6.0) ^b^, (6.6) ^c^, (5.86) ^d^	Cu	40.9
CaO	9.51 (5.9) ^a^, (5.9) ^b^, (2.4) ^c^, (7.07) ^d^	Ni	79.5
MgO	4.37 (1.7) ^a^, (1.7) ^b^, (1.83) ^c^, (1.28) ^d^	Cr	802.5
SO_3_	1.83 (0.13) ^a^, (0.13) ^b^, (0) ^c^, (0.15) ^d^	Hg	0.0
Na_2_O	0.29 (0.36) ^c^	As	62.8
K_2_O	1.00 (0.57) ^a^, (0.57) ^b^, (3.48) ^c^	Ba	69.0
P_2_O_5_	0.10 (0.74) ^a^, (0.74) ^b^, (0.12) ^c^, (0.96) ^d^	Sb	0.6
Loss to fire * * Determined at 1000 °C	2.59 (1.8) ^a^, (3.8) ^c^, (1.85) ^d^	Se	0.2

**^a^** See [43]. **^b^** See [44]. **^c^** See [13]. **^d^** See [45].

**Table 2:** Activity concentrations of radionuclides (Bq/kg) in the CBA-TB sample (activity concentrations of ^226^Ra, ^232^Th, and ^40^K were compared with the values from the literature (values in parentheses) and presented in the same table).

Radionuclide	^226^Ra	^232^Th	^40^K	^235^U	^238^U	^137^Cs	Gross Alpha Activity	Gross Beta Activity
CBA-TB	34 ± 2 (65.96) ^a^, (84) ^b^	20 ± 2 (96.5) ^a^, (79) ^b^	103 ± 10 (974) ^a^, (168) ^b^	0.8 ± 0.1	18 ± 4	<0.1	<154	<308

**^a^** See [60]. **^b^** See [61].

**Table 3:** The *R_max_* and *T_p_* values at various heating rates, are related to corresponding thermal decomposition regions of CBA-TB.

*β* (K min^−1^)	*R_max_* (%/min)			*T_p_* (°C)		
	I	II	III	I	II	III
10.3	1.935	3.186	0.996	82	374	670
20.9	3.573	6.905	2.065	94	378	686
32.1	4.424	11.473	3.277	100	382	690

**Table 4:** The kinetic parameters (*E_a_* and *A*) values are calculated by the standard method for corresponding reaction regions in the entire decomposition process of CBA-TB.

Method/ASTM E2890	Region		
Kinetic Parameters	I	II	III
*E_a_* (kJ·mol^−1^)	62.66 ± 0.51	490.28 ± 8.31	385.44 ± 9.60
*A* (s^−1^)	1.67 × 10^7^	9.48 × 10^37^	1.95 × 10^19^

**Table 5:** Values of thermodynamic parameters ΔH*, ΔG*, and ΔS*, which were calculated at *T_p_* values at the different heating rates (10.3 K/min, 20.9 K/min, and 32.1 K/min) for the corresponding reaction regions of decomposition process of CBA-TB.

Thermodynamic Parameters	Heating Rate, *β* (K min^−1^)	Region		
		I	II	III
ΔH* (kJ·mol^−1^)	10.3	59.71	484.90	377.60
	20.9	59.61	484.87	377.47
	32.1	59.56	484.83	377.43
**Average**		59.63	484.87	377.50
ΔG* (kJ·mol^−1^)	10.3	101.05	182.45	277.17
	20.9	102.45	180.58	275.46
	32.1	103.15	178.71	275.04
**Average**		102.22	180.58	275.89
ΔS* (J·mol^−1^·K^−1^)	10.3	−116.41	467.35	106.49
	20.9	−116.68	467.30	106.35
	32.1	−116.82	467.25	106.31
**Average**		−116.64	467.30	106.38

**Table 6:** The values of *Φ_o_*, A*, *E_a_*_,*c*_*,* and *σ* for the LogNormal *ddf* which describes complex reactivity during thermal conversion process of CBA-TB sample.

LogNormal *ddf* Parameters	
*Φ_o_* × 10^−4^ (mol (kJ)^−1^)	2.009 ± 0.004
A* (kJ mol^−1^)	2.302 ± 0.326
*E_a_*_,*c*_ (kJ mol^−1^)	337.23 ± 3.61
*σ* (kJ mol^−1^)	0.052 ± 0.012

**Table 7:** Comparison of the kinetic methods/models in regard to their fit to the measured TG-data for CBA-TB thermal conversion process.

Method/Model	Fit to	R^2^	Sum of Dev. Squares	Mean Residual (MR)	F-Test
Friedman	TG-signal	0.99983	22.591	0.109	1.028
Numerical	TG-signal	0.99983	21.976	0.101	1.000
Ozawa–Flynn–Wall	TG-signal	0.98598	1811.935	1.147	82.450
Kissinger–Akahira–Sunose	TG-signal	0.98968	1337.008	0.940	60.839
Vyazovkin	TG-signal	0.99977	29.300	0.120	1.333

**Table 8:** The values of *a*, *b*, *k_iso_*, and *T_iso_* were obtained from Constable plots (Figure 7) for the complex multi-step decomposition process of CBA-TB.

KCE-Branch	Δα	*a* (s^−1^)	*b* (mol·(kJ)^−1^)	*k_iso_* (s^−1^)	*T_iso_* (°C)
Region I	0.01–0.14	−5.09443 ± 0.15241	0.17048 ± 0.00212	8.04581 × 10^−6^	432.38
Region II	0.15–0.54	−1.88189 ± 0.2233	0.07626 ± 9.06241 × 10^−4^	0.01313	1304.07
Transition *	0.55–0.73	−43.07192 ± 3.87238	0.18758 ± 0.0112	8.47383 × 10^−44^	368.06
Region III	0.74–0.99	2.65488 ± 0.29398	0.04922 ± 2.58568 × 10^−4^	4.51731 × 10^2^	2170.55

**Table 9:** Kinetic parameters for p:, model scheme, which mechanistically describes the entire thermal decomposition process of CBA-TB.

p:, Model	
Model Scheme: A─B─C D─E─F G─H	
**Step: *A* → *B***	
**Reaction Type: A*n***	
Concentration equation ^a^: d(*a*→*b*)/d*t* = *A*·*n*·*a*·[−ln(*a*)][(n − 1)/n]·exp[−*E_a_*/(*RT*)]	
Activation energy (*E_a_*) (kJ·mol^−1^)	24.842
Log(PreExp), log*A*, *A* (s^−1^)	0.011
Dimension, *n*	0.652
Contribution	0.215
**Step: *B* → *C***	
**Reaction Type: F2**	
Concentration equation ^b^: d(*b*→*c*)/d*t* = *A*·*b*^2^·exp[−*E_a_*/(*RT*)]	
Activation energy (*E_a_*) (kJ·mol^−1^)	217.334
Log(PreExp), log*A*, *A* (s^−1^)	16.209
Contribution	0.181
**Step: *D* → *E***	
**Reaction Type: F*n***	
Concentration equation ^a^: d(*d*→*e*)/d*t* = *A*·*d*^n^·exp[−*E_a_*/(*RT*)]	
Activation energy (*E_a_*) (kJ·mol^−1^)	107.084
Log(PreExp), log*A*, *A* (s^−1^)	13.734
React. Order, *n*	1.975
Contribution	0.064
**Step: *E* → *F***	
**Reaction Type: R3**	
Concentration equation ^b^: d(*e*→*f*)/d*t* = *A*·3(*e*^(2/3)^)·exp[−*E_a_*/(*RT*)]	
Activation energy (*E_a_*) (kJ·mol^−1^)	30.830
Log(PreExp), log*A*, *A* (s^−1^)	−1.712
Contribution	0.304
**Step: *G* → *H***	
**Reaction Type: *Cnm***	
Concentration equation ^a,b^: d(*g*→*h*)/d*t* = *A*·*g^n^*·[1 + AutocatPreExp·*h^m^*]·Exp[−*E_a_*/(*RT*)]	
Activation energy (*E_a_*) (kJ·mol^−1^)	224.651
Log(PreExp), log*A*, *A* (s^−1^)	6.086
React. Order, *n*	6.392
Log(AutocatPreExp)	8.701
Autocat. Power, *m*	0.170
Contribution	0.236

**^a^** *a*, *d,* and *g* are concentrations of reactants. **^b^** *c*, *f,* and *h* are concentrations of products (*b* and *e* represent concentrations of intermediates).

**Table 10:** Conditions for adiabatic 24 (h) predictions using the Friedman’s (FR), numerical and p:, models in terms of the thermal safety of CBA-TB sample.

Method/Model	Friedman/Numerical/p:,
Enthalpy (Δ*H*) (J·g^−1^)	112.00
Specific heat (J·g^−1^·K^−1^)	5.00
*Phi* (*ϕ*) (dimensionless)	1.00
TMR adiabatic (h)	24.00
Temp. initial (°C)	21.96

## Data Availability

The data presented in this study are available on request from the corresponding author.

## Supplementary Materials

The following supporting information can be downloaded at: https://www.mdpi.com/article/10.3390/ma17235759/s1, Figure S1: “Match!” Phase Analysis Report integrated with XRD pattern graphics of the CBA-TB sample; Table S1: Kinetic model functions (in the differential form of the analytical kinetic functions, *f*(α)), used in the current work for computational procedure in the model-based kinetics analysis.; Supplementary Materials content: S.I. Model-free (isoconversional) kinetic approach (pages S-2–S-6); S.II. Model-based kinetic approach (pages S-6–S-9); S.III. Scale up—safety analysis: Characterization of runaway reactions and use of the kinetic data in adiabatic simulation prediction (pages S-9–S-12); Supplementary Materials Reference List: pages S-12–S-13.

## Nomenclature

***Abbreviations***	
IEA	International Energy Agency
EU	European Union
CCP	Combustion coal products
LOI	Loss of ignition
TEKO-B	Coal-fired power plant “Kostolac B”
MTSR	Maximum temperature of synthesis reaction
TMR	The time to the maximum rate
CBA	Coal bottom ash
CBA-TB	Coal bottom ash collected in coal-fired power plant “Kostolac B”
WD-XRF	Wavelength-dispersive equipped X-ray fluorescence
ICP-MS	Inductively coupled plasma mass spectrometry
HMs	Heavy metals
XRD	X-ray diffraction
XRPD	X-ray powder diffraction
IAEA	International Atomic Energy Agency
MARLAP	Multi-Agency Radiological Laboratory Analytical Protocols
TG	Thermogravimetry
DTG	Derivative thermogravimetry
FR	Friedman method
KAS	Kissinger-Akahira-Sunose method
OFW	Ozawa-Flynn-Wall method
VY	Vyazovkin method
KCE	Kinetic compensation effect
NLLS	The non-linear least square
ASTM	American Society for Testing and Materials
LEAF	Leaching Environmental Assessment Framework
IARC	International Agency for Research on Cancer
TA	Thermo-analytical
AED	The activation energy distribution
*ddf*	Density distribution function
MR	Mean residual
IKR	Isokinetic relationship
MW	Molecular weight
Δ*m_sample_*	the mass of powdered sample (mg)
*T*	the temperature (°C and/or K)
Δ*T*	the temperature range (interval) (°C)
*logA*	the logarithm of pre-exponential factor (-)
*A*	the pre-exponential factor (s^−1^)
*E_a_*	the activation energy (effective) (kJ mol^−1^)
Δ*m_res_*	the residual mass of the sample (expressed in%)
*T_p_*	the maximum (peak) temperature (°C)
*R_max_*	the maximum reaction rate (%/min)
*T_D24_*	initial temperature for an adiabatic process with time to the maximum rate equals to 24 h (°C)
ΔH*	changes in the activation enthalpy (kJ mol^−1^)
ΔS*	changes in the activation entropy (J mol^−1^ K^−1^)
ΔG*	changes in Gibbs free energy of activation (kJ mol^−1^)
*Φ(E_a_)_exp_*	experimentally obtained density distribution function of activation energies (mol (kJ)^−1^)
*Φ(E_a_)*	theoretically derived density distribution function (LogNormal) of activation energies (mol (kJ)^−1^)
*dE_a_*	infinitesimally small change of E_a_ (kJ mol^−1^)
Δ*E_a_*	the final change of E_a_, the E_a_ increment (kJ mol^−1^)
*Φ_o_*	the onset of the distribution (mol (kJ)^−1^)
*A**	the area of the distribution function (kJ mol^−1^)
*E_a,c_*	the central distribution value (median) (kJ mol^−1^)
*k_iso_*	the artificial isokinetic rate constant (s^−1^)
*T_iso_*	the artificial isokinetic temperature (°C)
*a*	the KCE constant _ intercept value (s^−1^)
*b*	the KCE constant _ slope value (mol (kJ)^−1^)
*T_exp_*	experimental temperature value (°C)
*E_a_*(α)	conversion dependent effective activation energy (kJ mol^−1^)
*A*(α)	conversion dependent pre-exponential factor (effective) (s^−1^)
[*E*, *A*, *f*(α)]	the kinetic triplet
*E*	the activation energy for the individual reaction step (kJ mol^−1^)
*A*	the pre-exponential factor for individual reaction step (s^−1^)
*n*	the reaction order
*m*	the autocatalysis reaction power exponent
*P*	the pressure (GPa)
*K_cat_*	the autocatalysis factor
*T**	the average temperature at maximum reaction rates, throughout all heating rates applied (°C and/or K)
d*T*/d*t*	the self-heat rate (K/s and/or K/min)
***Greek Letters***	
α	the conversion (the extent of reaction) (-)
*β*	the heating rate (K/min)
*γ*	the skewness
*θ*	the Bragg angle (°)
*λ*	the wavelength of Cu Ka radiation (Å)
*φ*	the gas flow rate (mL/min)
*σ*	the standard deviation (kJ mol^−1^)
*ϕ*	the thermal inertia factor (-)
