# Supplementary material for: Thermal Conversion of Coal Bottom Ash and Its Recovery Potential for High-Value Products Generation: Kinetic and Thermodynamic Analysis with Adiabatic TD24 Predictions
Source: Materials (Basel). 2024 Nov 25;17(23):5759. doi: 10.3390/ma17235759 (PMC11642072; doi:10.3390/ma17235759)
Supplement: Supplementary file 1 [file materials-17-05759-s001.zip › materials-3301008-supplementary.pdf]

Supplementary Material

Materials

Title: "**Thermal conversion of coal bottom ash and its recovery potential for high-value products generation: Kinetic and thermodynamic analysis with adiabatic T_{D24} predictions**"

Authors: Bojan Janković, Marija Janković* (*Corresponding author), Ana Mraković, Jelena Krneta Nikolić, Milica Rajačić, Ivana Vukanac, Nataša Sarap, Nebojša Manić

Corresponding author (*) *affiliation*: "Vinča" Institute of Nuclear Sciences – National Institute of the Republic of Serbia, University of Belgrade, Mike Petrovića Alasa 12-14, P.O. Box 522, 11001 Belgrade, Serbia

• Supplementary Material content (S-1):

S.I. Model-free (isoconversional) kinetic approach…………………………………………...S-2–S-6

S.II. Model-based kinetic approach…………………………………………………………..S-6–S-9

S.III. Scale up - safety analysis: Characterization of runaway reactions and use of the kinetic data in adiabatic simulation prediction……………………………………………………………..S-9–S-12

References……………………………………………………………………………………S-12–S13

• Supplementary Material - Results content (S-1):

»Match! Phase Analysis Report to X-ray Diffraction data related to CBA-TB sample………………...S–14

Figure S1. Match! Phase Analysis Report integrated with XRD pattern graphics of CBA-TB sample…………………………………………………………………………………………S-15

S.I. Model-free (isoconversional) kinetic approach

Based on the data provided by the TG measurements at different heating rates, kinetic models can be developed to accurately describe the decomposition process. Different established kinetic methods, such as Friedman (FR) [1], Kissinger-Akahira-Sunose (KAS) [2,3], Ozawa-Flynn-Wall (OFW) [4,5] and Vyazovkin (VY) [6], have been applied to find the model, which best fits the experimental data. All the mentioned methods are model-free approaches, which allow to determine the necessary parameters like activation energies and pre-exponential factors, without assumption of the reaction type. Therefore, the set of several TG measurements is required [7]. Model-free (isoconversional) methods are suitable for the investigation of multiple-step reactions, since in most cases, the decomposition reactions are usually multistep and not the first-order reactions.

To apply those methods, two assumptions are made. The first assumption of model-free analysis is that the reaction can always be described by the Eq. (S1). It was based on the dependence of the activation energy E_a and the pre-exponential factor A on the reaction progress α (conversion or the extent of conversion) as:

$$\frac{d\alpha}{dt} = A(\alpha)f(\alpha)e^{-\frac{E_\alpha(\alpha)}{RT}}, \quad \text{(S1)}$$

where $d\alpha/dt$ is the conversion rate of thermal decomposition, while $f(\alpha)$ represents the reaction (kinetic) model, and R is the universal gas constant (8.314 J·K^{-1}·mol^{-1}) (T is the temperature). The degree of conversion (or the conversion) can be obtained from the mass ratios at a given temperature or time, expressed as $\alpha = (m_o - m_T)/(m_o - m_f)$, where m_o is the initial mass, m_T is the mass obtained at the estimated temperature (or time), and m_f is the final mass at analyzed instant.

Secondly, it is assumed that the reaction rate at a constant conversion value can be described as a function, which is only dependent on the temperature.

The Ozawa-Flynn-Wall (OFW) method is an integral isoconversional approach, solving the Eq. (S1) by integration, which results in the Eq. (S2):

$$g(\alpha) = \int_0^{\alpha} \frac{d\alpha}{f(\alpha)} = \frac{A(\alpha)}{\beta} \int_{T_o}^{T} exp\left(-\frac{E_a(\alpha)}{RT}\right) dT, \tag{S2}$$

where this equation is transformed into Eq. (S3), by the approximation according to Doyle [8]:

$$log\beta = log\left(\frac{A \cdot E_a}{R \cdot g(\alpha)}\right) - 2.315 - 0.4567 \cdot \frac{E_a}{RT}. \tag{S3}$$

The graphic logarithm of the heating rate is applied to the inverse temperatures of the points with the same conversion at different heating rates, resulting in straight lines connecting points of the same conversion. From the slope of these lines the activation energy can be determined. The values of the pre-exponential factor are subsequently determined from the kinetic compensation effect (KCE) [9].

The Kissinger-Akahira-Sunose (KAS) is based on the Eq. (S2), but using the Coats-Redfern temperature integral approximation [10], which modify an above-indicated equation into the Eq. (S4) adapted for isoconversional calculations, as:

$$ln\left(\frac{\beta}{T^2}\right) = ln\left[\frac{A \cdot R}{E_a \cdot g(\alpha)}\left(1 - \frac{2RT}{E_a}\right)\right] - \frac{E_a}{RT}. \tag{S4}$$

The activation energy can be calculated from the plot of the logarithm of $\ln(\beta/T^2)$ against $(1/T)$, taking into account that $2RT/E_a \ll 1$, for the temperature range considered. The values of the pre-exponential factor are determined from the kinetic compensation effect (KCE), indicated previously. The KAS method is based on the temperature integral approximation using $p(x) = e^{-x}/x^2$ (where $x = E_a/RT$), for the x range of $20 \leq x \leq 50$.

The Friedman (FR) approach relies on a differential solution of the Eq. (S1), with assumption that the chemistry of the decomposition process depends only on the rate of mass loss and is independent from the temperature. The activation energy and the pre-exponential factor are determined analog to the previous established methods, by the slope and intercept of the straight lines, connecting points of the same conversion, when plotting the logarithm of the conversion rate against the inverse temperatures, such as:

$$ln\left(\frac{d\alpha}{dt}\right) = ln[A \cdot f(\alpha)] - \frac{E_a}{RT}. \tag{S5}$$

Vyazovkin (VY) method uses the non-linear regression proposed by Senum and Yang, which makes it more accurate over a wider range of TG-data [11], and circumvents the inaccuracies related to the analytical approximation of the temperature integral. However, its application remains limited as mass transfer becomes limiting, at very high conversions (above ~ 80 %).

Vyazovkin's (VY) isoconversional (advanced) method is the widely recommended integral approach, for the accurate determination of activation energies, E_a [12]. The VY method proposes an exact equation (the non-linear) based on the general assumption, that the reaction (kinetic) model is independent on the heating rate. The activation energy at a specific conversion value, α, is obtained by determining the E_a value, that minimizes the Eq. (S6):

$$\sum_{i=1}^{n}\sum_{j\neq i}^{n}\frac{I(E_{a,\alpha},T_{\alpha,i})\cdot\beta_j}{I(E_{a,\alpha},T_{\alpha,j})\cdot\beta_i}=min. \tag{S6}$$

where

$$I(E_{a,\alpha},T_\alpha)=\int_0^{T_\alpha}e^{-\frac{E_{a,\alpha}}{RT}}dT. \tag{S7}$$

In above equations, $E_{a,\alpha}$ and T_α are the activation energy and the temperature at a conversion α, respectivelly, obtained from independent experimental runs *i* and *j*, and performed at different heating rates, β's. The integral is numerically evaluated by using the trapezoidal rule and a uniform grid spacing, that is continuaslly decreased, until a difference in the integral values is smaller than 10^{-6}, between the consecutive interactions, is obtained. Different activation energy values are then used in above equations and, the activation energy for the process, determined as the value of $E_{a,\alpha}$, which gives the lower result, for the Eq. (S6). For all calculations, the sophisticated software NETZSCH Kinetics Neo (Product version 2.7.3.15, Build date 6/11/2024) was used. This is a multi-purpose software used to save costs and time, and also to simplify kinetic calculations, which are based on previous knowledge of decomposition characteristics of considered material. The software allows the kinetic study by using thermal analysis techniques, such as TG, DTG and/or DSC. For established isoconversional methods, in order to estimate the pre-exponential factors as log$A$ values (software has ability to transforms "*ln*"

scale into "*log*" scale data, where normally operates), the actual software uses the kinetic compensation effect (KCE) model, which was previously indicated. The reliability of the modeling using the Kinetics Neo software relies on the accurate and reproducible thermal analysis data, which enables the numerical optimization of the investigated process, and represents the model-free method (being used FR results), using the non-linear least square optimization. Model-free (isoconversional) and numerical optimization methods do not require knowledge of the analytical form of the reaction model.

After the implementation of model-free kinetic approach, the numerical (model-free) optimization was applied to TG-signal results, by minimizing the objective function, Ω, as:

$$\Omega = \sum_{j=1}^{N} \sum_{k=First_j i}^{Last_j} w_{j,k} \left(TG_{exp_{j,k}} - TG_{calc_{j,k}} \right)^2 \quad \text{(S8)}$$

$$w_j = \frac{1}{|(TG_{max})|_j + |(TG_{min})|_j} \quad \text{(S9)}$$

where N is the number of measurements, j is the index of the given measurement at i-th heating rate, $First_j$ is the index of the first point of a given curve, $Last_j$ is the index of the last point of a given curve, $TG_{exp_{j,k}}$ is the experimental (measured) value of the point k of the curve j, $TG_{calc_{j,k}}$ is the calculated (simulated) value of the point k of the curve j, and w_j is the weighting factor, for the observed curve j. Numerical optimization method is the model-free method using non-linear least square optimization from Friedman's (FR) model-free results, minimizing the sum of squared residue (Ω). This approach was implemented in the Kinetics Neo software. Numerical method searches optimal functions $E_a(\alpha)$ and $logA(\alpha)$, in order to obtain a best fit for the conversion. The results of model-free method (curves $E_a(\alpha)$ and $A(\alpha)$) then optimized numerically, in order to achieve the better fit between the experimental and the simulated curves. The function for optimization is the sum of squares of deviations between measured and simulated TG-curves (Eq. (S8)). This sum is calculated over all curves and all points in each thermo-analytical (TA) curve. Therefore, the numerical method searches numerically values of $E_a(\alpha)$ and $logA(\alpha)$, which minimize the optimization function, Ω; internally, each point of curves $E_a(\alpha)$ and $A(\alpha)$ is the subject of small changes, and for each change, the sum of squares of residuals is checked: namely, is it better or worse than before. If the better, than new point in $E_a(\alpha)$ or $A(\alpha)$ is then saved. The

iterations are repeated until no any numerical improvements happen. Advantage of the numerical optimization is reflected in the fact that can be applied for multiple-step reactions with evaluation of each reaction point at various heating rates, where mean values of kinetic parameters are also known.

S.II. Model-based kinetic approach

In addition to described model-free methods, a model-based approach was applied for analyzing the non-isothermal decomposition kinetics of tested coal bottom ash sample. Model-based approach uses model-free (isoconversional) analysis results, in order to determine initial kinetic decomposition parameters, and gain insight regarding the number of steps involved, and types of kinetic models which govern the process. It uses multivariate non-linear regression method (MVarNLRM) for resolving the concentration equations in multi-stage process. Kinetics Neo software includes a set of basic equations describing macro-kinetics of the process which is analyzed. Each stage of the process can correspond to one (or several) of the equations listed in Table S1. The type of $f(\alpha)$ function depends on the nature of the process, and is usually selected *a priori*. For the user's convenience, the notation of parameters and variables in Table S1 is the same as in the Kinetics Neo software. Software uses p parameter, which corresponds to the conversion, $p = \alpha$, and parameter $e = 1 - \alpha$ (the remaining fraction unreacted). The model-based analysis assumes that reaction comprises several elementary reaction steps, which can be a series of the consecutive reactions.

Table S1. Kinetic model functions (in the differential form of the analytical kinetic functions, $f(\alpha)$) used in the current work, for computational procedure in the model-based kinetics analysis.

**Model**	**Symbol**	***f*(α)**
Phase boundary-controlled reaction (contracting disk, 1D)	R1/F0	$(1 - \alpha)^0$
Phase boundary-controlled reaction (contracting area, 2D)	R2	$2\cdot(1 - \alpha)^{1/2}$

Phase boundary-controlled reaction (contracting volume, 3D)	R3	$3\cdot(1 - \alpha)^{2/3}$
Random nucleation, unimolecular decay law, first order chemical reaction	F1	$(1 - \alpha)$
Second order chemical reaction	F2	$(1 - \alpha)^2$
n-th order chemical reaction ($n \neq 1$)	F*n*	$(1 - \alpha)^n$
Two-dimensional growth of nuclei (Avrami equation)	A2	$2\cdot(1 - \alpha)[- \ln(1 - \alpha)]^{1/2}$
Three-dimensional growth of nuclei (Avrami equation)	A3	$3\cdot(1 - \alpha)[- \ln(1 - \alpha)]^{2/3}$
n-dimensional nucleation (Avrami-Erofeev equation)	A*n*	$n\cdot(1 - \alpha)[- \ln(1 - \alpha)]^{1-1/n}$
One-dimensional diffusion, parabola law	D1	$1/2\alpha$
Two-dimensional diffusion, Valensi equation	D2	$1/[- \ln(1 - \alpha)]$
Three-dimensional diffusion, Jander equation	D3	$(3/2)(1 - \alpha)^{2/3}/[1 - (1 - \alpha)^{1/3}]$
Three-dimensional diffusion, Ginstling-Brounstein	D4	$(3/2)/[(1 - \alpha)^{-1/3} - 1]$
Prout-Tompkins equation	B1	$(1 - \alpha)\cdot\alpha$
Expanded Prout-Tompkins equation	B*na*	$(1 - \alpha)^n\cdot\alpha^a$
First order with autocatalysis	C1	$(1 + k_{cat}\cdot\alpha)(1 - \alpha)$
n-th order with autocatalysis	C*n*	$(1 + k_{cat}\cdot\alpha)(1 - \alpha)^n$
n-th order and *m*-power with autocatalysis	C*nm*	$(1 - \alpha)^n\cdot\alpha^m$
Expanded Šestak-Berggren (SB) equation	SB*nmq*	$(1 - \alpha)^n\cdot\alpha^m\cdot[-\ln(1 - \alpha)]^q$
Kamal-Sourour equation	KS	$(k_1 + k_2\cdot\alpha^m)(1 - \alpha)^n$
Nakamura crystallization	Nk (A*n* + H-L)	$f(\alpha)\cdot K(T)$, $f(\alpha) = n\cdot(1 - \alpha)[- \ln(1 - \alpha)]^{1-1/n}$, where for analytical dependence of the rate constant $K(T)$, Hoffman-Lauritzen (H-L) theory is used (non-Arrhenius).

Šestak-Berggren crystallization or Sbirrazzuoli crystallization	(SBC/SC) (SB + H-L)	$f(\alpha)\cdot K(T)$, $f(\alpha) = (1 - \alpha)^n \cdot \alpha^m \cdot [-\ln(1 - \alpha)]^q$, where for analytical dependence of the rate constant $K(T)$, Hoffman-Lauritzen (H-L) theory is used (non-Arrhenius)

The reaction rate for the individual reaction steps can be expressed through the Eq. (S10) [13]:

$$Reaction\ rate_j = A_j \cdot f_j(e_j, p_j) \cdot e^{-\frac{E_j}{RT}}, \tag{S10}$$

where $f_j(e_j,p_j)$ represents the function of reaction type, e_j is the initial reactant concentration, p_j is the product concentration, A_j is the pre-exponential factor, E_j is the activation energy, while j represents the number of specific reaction steps. This approach takes into account certain assumptions such as: a) the process has to consist of a number of reaction steps, and for each of these steps the reaction rate can be described by the Eq. (S10), depending on the concentration of the initial reactant, e_j, the concentration of the product, p_j, the pre-exponential factor, A_j, and the activation energy, E_j, b) the relevant factors including activation energy, pre-exponential factor, order of reaction, and reaction type for each step, are considered to be constant, and c) it is assumed that the thermo-analytical (TA) signal equals the sum of the signals of the single reaction steps, while the effect of each step is calculated by a multiplying the reaction rate with the mass loss of this step, expressed through the following equation:

$$m = m_o - \Delta m \cdot \left[\sum_{j=1}^{n} Contribution_j \int \left(\frac{d(x_j \to y_j)}{dt}\right)_j dt\right], \tag{S11}$$

where m is the mass, m_o is the initial mass, Δm is the total mass change), while $(x_j - y_j)$ represents the heat flow on the reaction path from the reactant "x" to the product "y", and "$Contribution_j$" corresponds to the contribution of the "j" reaction step to the overall heat flow. For model-based method, the change of kinetic mechanism is simulated by several reaction steps, with own kinetic triplets (A_j, E_j, f_j), ranked in a specific reactions order, through the constructed mechanistic (reaction) scheme, which has special

marking in the computational software (the kinetic scheme code designation), depending on whether the branching exists or not. Model-based kinetic analysis is powerful tool that allows accurate estimation of number of steps, their contribution to the total effect of the reaction or about reaction order (or kinetic exponents) for each considered step.

S.III. Scale up - safety analysis: Characterization of runaway reactions and use of the kinetic data in adiabatic simulation prediction

The precise prediction of reaction progresses in adiabatic conditions is necessary for the safety analysis of many technological processes [14-18]. Calculations of an adiabatic temperature-time curve for the reaction progress can also be used to determine the decrease of the thermal stability of materials during storage at temperatures near the threshold temperature for triggering the reaction. Due to insufficient thermal convection and limited thermal conductivity, a progressive temperature increase in the sample can easily take place, resulting in hazard situation. Because decomposition reactions usually have a multi-step nature, the accurate determination of the kinetic characteristics strongly influences the ability to correctly describe the progress of the process. The use of simplified kinetic models for the assessment of runaway reactions can, on one hand, lead to economic drawbacks, since they result in exaggerated safety margins. On the other hand, it can cause the dangerous situations, when the heat accumulation is underestimated. For adiabatic self-heating reactions, incorrect kinetic description of the process is usually the main source of prediction errors. The kinetic based approach for determination of the time to maximum rate under adiabatic conditions can be seen elsewhere [19].

An important problem with adiabatic data is the uncertainty of the reactant state at the onset temperature of the adiabatic mode. The problem originates from the simple facts that it is just impossible to maintain adiabatic conditions from the beginning of an experiment and the sensitivity of an adiabatic calorimeter is limited. It is common practice to apply one or another type of thermal initiation. The adiabatic mode is established when a calorimeter first detects heat generation by the reaction. Usually, it is assumed that due to high sensitivity of the calorimeter, the conversion at the

detected onset temperature is negligible (*apropos* "zero" assumption), although, it is evident that reaction started before the onset and conversion is non-zero, but there is no way to determine it directly. Nevertheless, it turns out that even very small but non-zero conversion strongly affects the reaction course and kinetics evaluated under "zero" assumption, may be unsafe or even completely wrong. Consequently, this description is strongly grounded on the fact that the established complex kinetic mechanism (determined by the model-based approach) contains reactions described by n-th order reaction and autocatalytic reaction. Formulation of adiabatic problem in the actual simulation that operates through the Kinetic Neo software prediction tool was based on the concept presented by Kossoy et al. [20]. In this work, DSC measurements or other calorimetry testing procedures were not directly implemented, but the given software is capable of simulating the process in the described reaction (adiabatic) conditions, based on the reported kinetic results using both, model-free and model-based methods, respectively. So in that sense, an experimental data cannot be directly used to calculate the time for the reaction and the adiabatic temperature rise. The experimental data must be corrected for the effect of the vessel's heat capacity using the *phi* factor (ϕ). There are several methods of correcting for the *phi* available in the literature. The most straightforward is to correct the measured adiabatic temperature rise by the following:

$$(\Delta T_{ad})_{actual(real)} = \phi(\Delta T_{ad})_{measured}, \tag{S12}$$

and the time for the reaction by

$$t_{actual(real)} = \frac{t_{measured}}{\phi}. \tag{S13}$$

Alternative methods of correcting for *phi* given in the literature are the method by Fisher presented in the DIERS project manual [21] and that by Huff [22]. All these methods can be compared with an ideal case. The ideal case was generated from TA data by kinetics results, such that when the simulation model was run with relevant *phi* factor, the simulation model reproduced exactly the experimental curve. Having exactly matched the experimental data with the simulation curve, the simulation model

was then run with ϕ = 1 (the lower is the influence of the container, and the more representative the result is of the sample itself). Sensitivity analysis related to time to runaway includes important physical data, such as pre-exponential factor, activation energy, heat capacity and start (initial) temperature for the reaction, and they can be accurately determined based on the obtained models, used in the simulation program. So that, for the achievement of the adiabatic conditions leads to the following equation:

$$\frac{dT}{dt} = \frac{1}{\phi} \cdot \Delta T_{ad,real} \cdot \left(\frac{d\alpha}{dt}\right), \tag{S14}$$

where dT/dt is the self-heat rate corresponding to T, $\Delta T_{ad,real}$ is the adiabatic temperature rise expressed as $\Delta T_{ad,real} = \Delta H/c_p$ (where ΔH is the enthalpy ($J \cdot g^{-1}$) and c_p is the heat capacity of the material ($J \cdot g^{-1} \cdot K^{-1}$)), while (d$\alpha$/d$t$) represents kinetic expression described by the Eq. (S1) in the case of isoconversional analysis, i.e., described by Eq. (S10), in the case of the model-based analysis. One can now use the kinetic based approach for the prediction of the reaction progress $\alpha(t)$ and the rate dα/dt, as well as the development of the temperatures $T(t)$, and dT/dt and adiabatic induction times, at any selected starting temperatures. It should be noted that the ϕ factor influences the simulation of the process in Accelerating Rate Calorimeter (ARC) experiment, so, the ϕ factor influences on the following:

a) $\Delta T_{ad,measured}$, because it comes from above description that $\Delta T_{ad,measured} = (1/\phi) \cdot \Delta T_{ad,real}$, and
b) Time to maximum rate (TMR) (the time from the beginning of an adiabatic process to the maximum reaction rate), in a different level depending on the type of decomposition kinetics.

The ϕ factor (thermal inertia factor) represents the ratio of the heat capacity of the material and the vessel to the heat capacity of the material c_p. For the absence of container, ϕ = 1. So, the thermal Inertia (ϕ factor) can be presented by the following expression:

$$\phi = 1 + \frac{(Mass \times Specific\ heat\ capacity)_{Container}}{(Mass \times Specific\ heat\ capacity)_{Sample}}. \tag{S15}$$

Therefore, when the ϕ factor is close or equal to unity, this represents a strict adiabatic conditions.

For the complex reactions, the complete kinetic model comprising heat balance equation and energy source equation, which are integrated numerically within the Kinetics Neo software prediction tool, resulting in the $T(t)$ response or in the $\alpha(t)$ response. Likewise, the software enables direct estimation of TMR (under adiabatic condition) and T_{D24} quantity, where the latter represents the temperature at which the time to maximum rate of the runaway reaction is 24 hours (in other words, T_{D24} means that an intervention is possible within 24 hours).

Considering industrial scale-up process, the knowledge of the enthalpy value alone is not always enough for a safe chemical process. If cooling fails, the continuing reaction will increase the temperature in the reactor until the reactants are consumed. Then, the reaction and corresponding self-heating will have finished and the final theoretical temperatures will be achieved. This temperature is called the maximum temperature of synthesis reaction (MTSR). Safety of industrial processes depends on how high the MTSR is. If it is too high, it can initialize a secondary process with further self-heating. This secondary reaction is usually some decomposition reaction, which is exothermal in its nature, and may leads to a further temperature increase. In fact, if the rapid secondary reaction is initialized, the risk of runaway and thermal hazard is then very high. If the value of the MTSR is lower than T_{D24}, this means that after finishing the primary reaction, the rapid secondary reaction is not initialized, and the risk of runaway reaction is the very low. If the MTSR is higher than T_{D24}, the secondary reaction starts already during the primary reaction, and it is impossible to avoid the runaway, with an accompanying dangerous consequences.

References

1. Friedman, H.L. Kinetic of thermal degradation of char-forming plastics from thermogravometry. Application to a phenolic plastic. *J. Polym. Sci. Polym. Symp*. **1964**, *6*, 183-195. https://doi.org/10.1002/polc.5070060121
2. Kissinger, H.E. Reaction kinetics in differential thermal analysis. *Anal. Chem*. **1957**, *29*, 1702-1706. https://doi.org/10.1021/ac60131a045
3. Akahira, T.; Sunose, T. Method of determining activation deterioration constant of electrical insulating materials. *Rep. Res. Chiba Inst. Technol*. (*Sci. Technol*.), **1971**, *16*, 22-31.
4. Ozawa, T. A new method of analyzing thermogravimetric data. *Bull. Chem. Soc. Jpn*. **1965**, *38*, 1881-1886. https://doi.org/10.1246/bcsj.38.1881

5. Flynn, J.H.; Wall, L.A. General treatment of the thermogravimetry of polymers. *J. Res. Natl. Bur. Stand. A Phys. Chem*. **1966**, *70A*, 487-523. doi: 10.6028/jres.070A.043
6. Vyazovkin, S.; Dollimore, D. Linear and nonlinear procedures in isoconversional computations of the activation energy of nonisothermal reactions in solids. *J. Chem. Inf. Comput. Sci*. **1996**, *36*, 42-45. http://dx.doi.org/10.1021/ci950062m
7. Vyazovkin, S. Model-free kinetics - Staying free of multiplying entities without necessity. *J. Therm. Anal. Calorim*. **2006**, *83*, 45-51. https://doi.org/10.1007/s10973-005-7044-6
8. Doyle, C. Series approximations to the equation of thermogravimetric data. *Nature* **1965**, *207*, 290-291. https://doi.org/10.1038/207290a0
9. Vyazovkin, S.; Linert, W. The application of isoconversional methods for analyzing isokinetic relationships occurring at thermal decomposition of solids. *J. Solid State Chem*. **1995**, *114*, 392-398. https://doi.org/10.1006/jssc.1995.1060
10. Coats, A.W.; Redfern, J.P. Kinetic parameters from thermogravimetric data. *Nature* **1964**, *201*, 68-69. https://doi.org/10.1038/201068a0
11. Pérez-Maqueda, L.; Criado, J. The accuracy of Senum and Yang's approximations to the Arrhenius integral. *J. Therm. Anal. Calorim*. **2000**, *60*, 909-915. https://doi.org/10.1023/A:1010115926340
12. Vyazovkin, S. Evaluation of activation energy of thermally stimulated solid-state reactions under arbitrary variation of temperature. *J. Comput. Chem*. **1997**, *18*, 393-402. https://doi.org/10.1002/(SICI)1096-987X
13. Moukhina, E. Initial kinetic parameters for the model-based kinetic method. *High Temp. High Press*. **2013**, *42*, 287-302. ISSN 0018-1544
14. Frank-Kamenetskii, D.A. *Diffusion and Heat Transfer in Chemical Kinetics*, Plenum Press, New York, London, 1969.
15. Dien, J.M.; Fierz, H.; Stoessel, F.; Killé, G. The thermal risk of autocatalytic decompositions: A kinetic study. *Chimia* **1994**, *48*, 542-550. https://doi.org/10.2533/chimia.1994.542
16. Pastré, J.; Wörsdörfer, U.; Keller, A.; Hungerbühler, K. Comparison of different methods for estimating TMR_{ad} from dynamic DSC measurements with ADT 24 values obtained from adiabatic Dewar experiments. *J. Loss Prev. Process Ind*. **2000**, *13*, 7-17. https://doi.org/10.1016/S0950-4230(99)00061-3
17. Ticmanis, U.; Pantel, G.; Wilker, S.; Kaiser, M. Precision required for parameters in thermal safety simulations. In Proceedings of the 32nd International Annual Conference of ICT, Karlsruhe, Germany, 3-6 July 2001; p. 135.
18. Folly, P. Thermal stability of explosives. *Chimia* **2004**, *58*, 394-400. ISSN 0009-4293, https://doi.org/10.2533/000942904777677759
19. Roduit, B. Advanced kinetics-based simulation method for determination of the thermal aging, thermal runaway TMR_{ad} and SADT using DSC. Analysis Report (example) (The AKTS AG Advanced Kinetics and Technology Solutions, http://www.akts.com), June 11th, 2008, pp. 22-29.
20. Kossoy, A.; Misharev, P.; Belochvostov, V. Peculiarities of Calorimetric Data Processing for Kinetics Evaluation in Reaction Hazard Assessment. Presented at 53rd Annual Calorimetry Conference, Midland, Michigan, USA, 1998, pp. 1-9.
21. Fisher, H.G.; Forrest, H.S.; Grossel, S.S.; Huff, J.E.; Muller, A.R.; Noronha, J.A.; Shaw, D.A.; Tilley, B.J. Emergency Relief System Design Using DIERS Technology: The Design Institute for Emergency Relief Systems (DIERS) Project Manual, 1st Edition, Wiley-AIChE, Hoboken, New Jersey, USA, 1993, pp. 1-576. ISBN: 978-0-816-90568-3, doi:10.1002/9780470938317.
22. Huff, J.E. Emergency venting requirements. *Plant/Operat. Prog*. **1982**, *1*, 211-229. https://doi.org/10.1002/prsb.720010405

● Match! Phase Analysis Report

Institute of Technical Science of SASA Sample: CBA-TB_1.0_origin

Sample Data

File name: CBA-TB_1.0_za origin.dat

Data range: 10.050º - 80.050º

Number of points: 7001

Step size: 0.010

Rietveld refinement converged: No

Alpha2 subtracted: No

Background subtr.: No

Data smoothed: No

2theta correction: 0.05º

Radiation: X-rays

Wavelength: 1.541874 Å

Matched Phases

Index	***Amount (%)***	***Name***	***Formula sum***
A	45.9	Anorthite	$CaAl_2Si_2O_8$
B	27.0	Muscovite	$Al_{2.8}Fe_{0.1}H_2K_{0.6}Mg_{0.04}Na_{0.37}O_{12}Si_{3.04}Ti_{0.02}$
C	20.6		SiO_2
D	3.5	Periclase	MgO
E	2.9	Iron(III) oxide Hematite	Fe_2O_3

A: Anorthite (45.9 %), B: Muscovite (27.0 %), C: SiO_2 (20.6 %), D: Periclase (3.5 %)

E: Iron(III) oxide Hematite (2.9 %)

Diffraction Pattern Graphics

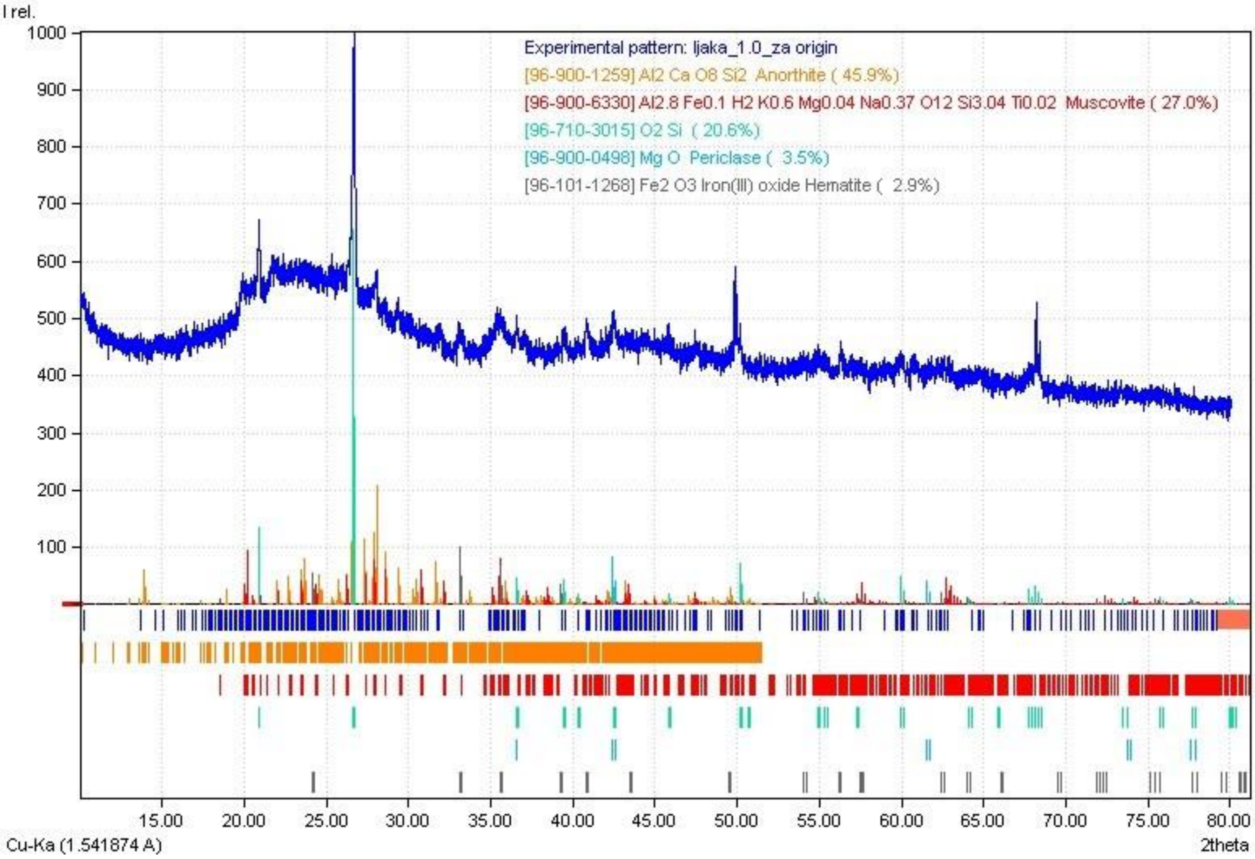

Reference: Match! is an easy-to-use software for phase analysis using powder diffraction data (Version 3.10.2) by CRYSTAL IMPACT, K. Brandenburg & H. Putz GbR Postfach 1251 D-53002 Bonn, Germany

Figure S1. Match! Phase Analysis Report integrated with XRD pattern graphics of CBA-TB sample.
